# Induction of Functional Specific Antibodies, IgG-Secreting Plasmablasts and Memory B Cells Following BCG Vaccination

**DOI:** 10.3389/fimmu.2021.798207

**Published:** 2022-01-05

**Authors:** Julia Bitencourt, Marco Polo Peralta-Álvarez, Morven Wilkie, Ashley Jacobs, Daniel Wright, Salem Salman Almujri, Shuailin Li, Stephanie A. Harris, Steven G. Smith, Sean C. Elias, Andrew D. White, Iman Satti, Sally S. Sharpe, Matthew K. O’Shea, Helen McShane, Rachel Tanner

**Affiliations:** ^1^ The Jenner Institute, Nuffield Department of Medicine, University of Oxford, Oxford, United Kingdom; ^2^ Laboratório Avançado de Saúde Pública, Instituto Gonçalo Moniz, Fundação Oswaldo Cruz (IGM/Fiocruz), Salvador, Brazil; ^3^ Department of Medicine, Institute of Infectious Diseases and Molecular Medicine, University of Cape Town, Cape Town, South Africa; ^4^ Department of Infection Biology, Faculty of Infectious and Tropical Diseases, London School of Hygiene and Tropical Medicine, London, United Kingdom; ^5^ Division of Biosciences, Brunel University, London, United Kingdom; ^6^ United Kingdom Health Security Agency, Porton Down, Salisbury, United Kingdom; ^7^ Institute of Immunology and Immunotherapy, College of Medical and Dental Sciences, University of Birmingham, Birmingham, United Kingdom

**Keywords:** antibodies, B cells, humoral immunity, tuberculosis, TB, BCG, vaccine

## Abstract

Tuberculosis (TB) is a major global health problem and the only currently-licensed vaccine, BCG, is inadequate. Many TB vaccine candidates are designed to be given as a boost to BCG; an understanding of the BCG-induced immune response is therefore critical, and the opportunity to relate this to circumstances where BCG does confer protection may direct the design of more efficacious vaccines. While the T cell response to BCG vaccination has been well-characterized, there is a paucity of literature on the humoral response. We demonstrate BCG vaccine-mediated induction of specific antibodies in different human populations and macaque species which represent important preclinical models for TB vaccine development. We observe a strong correlation between antibody titers in serum versus plasma with modestly higher titers in serum. We also report for the first time the rapid and transient induction of antibody-secreting plasmablasts following BCG vaccination, together with a robust and durable memory B cell response in humans. Finally, we demonstrate a functional role for BCG vaccine-induced specific antibodies in opsonizing mycobacteria and enhancing macrophage phagocytosis *in vitro*, which may contribute to the BCG vaccine-mediated control of mycobacterial growth observed. Taken together, our findings indicate that the humoral immune response in the context of BCG vaccination merits further attention to determine whether TB vaccine candidates could benefit from the induction of humoral as well as cellular immunity.

## 1 Introduction

Tuberculosis (TB), caused by *Mycobacterium tuberculosis* (*M.tb*), remains a major global health threat with 9.9 million new cases and 1.5 million deaths in 2020 (1). There are currently no validated correlates of protection from TB. However, *M.tb* is an intracellular pathogen and the necessity for a T cell response in conferring acquired immunity to TB has been demonstrated in numerous studies (2–6). This has led to limited attention on the humoral response to TB, although emerging evidence suggests that antibodies may play a more significant role in protection than previously appreciated (7–10). Antibodies could contribute to protection directly through increasing phagocytosis and phagolysosome formation or bacterial neutralization, and/or indirectly through enhancing T cell-mediated immunity (7). It has recently been shown that compared to antibodies from patients with active TB disease (ATB), antibodies from individuals with latent TB infection (LTBI) have unique Fc functional profiles, selective binding to FcγRIII and distinct antibody glycosylation patterns, and also that they drive enhanced phagolysosomal maturation, inflammasome activation and macrophage killing of intracellular *M.tb* (9).

Bacillus Calmette Guérin (BCG) is the only currently available vaccine against TB. BCG confers incomplete and variable protection against pulmonary TB in adolescents and adults, and a new more efficacious TB vaccine is needed (11, 12). However, it is uncertain which aspects of the immune response a candidate vaccine should aim to induce in order to confer protection that is superior to BCG. Due to the important role of BCG vaccination in protecting infants from severe forms of TB disease, and its potential non-specific effects protecting from all-cause mortality, most TB vaccine candidates are designed as a heterologous boost to a BCG prime (13, 14). It is thus critical to understand the immune response to BCG vaccination and which aspects will be, or would ideally be, induced or boosted. Furthermore, because BCG vaccination is partially protective, and can confer superior protection when administered intravenously in macaques (15, 16) or against sustained *M.tb* infection when administered as a revaccination in non-LTBI South African adolescents (17), studying the immune response to BCG offers a valuable opportunity to explore immune mechanisms of protection and apply these to inform the design of more efficacious TB vaccine candidates.

Robust Th1 responses to BCG vaccination have been described and are generally considered to be essential, but not sufficient, for protection (18–21). A role for trained innate immunity, unconventional T cells and humoral immunity in BCG-mediated protection has been proposed (22–24). Indeed, in a *post-hoc* correlates of risk analysis, levels of Ag85A-specific IgG were associated with reduced risk of TB disease in BCG-vaccinated South African infants (25). We have comprehensively reviewed what is currently known about the humoral immune response to BCG vaccination and revaccination across species (26). In brief, the literature presents inconsistent evidence for the induction of specific antibody responses following BCG vaccination, and the relevance of these responses is unclear, with support both for (25, 27–30) and against (28, 31–33) a protective function. Interestingly, recent evidence suggests that BCG vaccination can modulate antibody glycosylation patterns with potential relevance for functionality (34), and IgM titers were among the strongest markers of reduced bacterial burden following *M.tb* challenge of BCG immunized macaques (35).

Antibodies are produced by antibody-secreting B cells (ASCs). Upon activation by recognition of their cognate antigen during infection or vaccination, B cells undergo clonal expansion and differentiate into plasmablasts and memory B cells (mBCs) (36). While plasmablasts and plasma cells secrete antibody, mBCs can survive quiescently for decades, poised to rapidly respond to antigen re-stimulation by differentiating into short- and long-lived ASCs that can prolong the duration of high serum antibody levels (37, 38). It is generally accepted that mBC-derived ASCs are central to the long-term protection mediated by most licensed vaccines (36, 39), and the dynamics, magnitude and specificity of the ASC response to vaccination against other infections have been described (40–42). While one study has reported a significantly higher frequency of purified protein derivative (PPD)-specific mBCs in peripheral blood mononuclear cells (PBMC) from historically BCG-vaccinated compared with unvaccinated volunteers (43), the longitudinal ASC response following BCG vaccination has not been described. Evaluating these cells may provide a more dynamic, discriminative measure of humoral immunity than detecting stable serum antibodies by conventional serology.

The hitherto poorly-defined humoral response to BCG vaccination merits further investigation. We set out to explore the BCG vaccine-induced specific antibody response in serum collected from different cohorts of human volunteers representing TB-endemic and non-endemic populations. As the macaque model is widely used in TB studies (44), we included macaque samples to better understand the translatability of antibody responses to humans. While serum is the most widely-used sample for the assessment of antibody responses, it is often a limited resource, and we therefore explored in both species whether plasma can be used interchangeably. We also sought to determine the frequency and kinetic of BCG-induced antigen-specific ASCs (both plasmablasts and mBCs), and their association with serum antibody levels. Finally, we employed functional assays to explore a potential role for antibodies *in vitro*. Our findings offer proof-of-concept and provide some logistical foundations for a more comprehensive characterization of the BCG vaccine-induced antibody response across different populations and vaccine regimens for which different levels of protection are observed.

## 2 Methods

### 2.1 Samples

#### 2.1.1 Human Samples

Human PBMC, serum and plasma samples were taken from a randomized controlled clinical study of healthy BCG-naïve UK adults, henceforth referred to as human Study 1. Volunteers were randomized to receive intradermal (ID) BCG SSI at a standard dose (2-8 x 10^5^ CFU) (n=27) or to be unvaccinated controls (n=8). Samples were used from baseline and follow-up visits at 7, 14, 21, 28 and 84 days. ELISA operators were blinded to treatment group. The study was approved by the NHS Research Ethics Service (NRES) Committee South Central - Oxford B (REC reference 15/SC/0022), and registered with ClinicalTrials.gov (NCT02380508). All aspects of the study were conducted according to the principles of the Declaration of Helsinki and Good Clinical Practice. Volunteers provided written informed consent prior to screening. Baseline biochemical and hematological analysis and serological testing for human immunodeficiency virus (HIV), hepatitis B virus (HBV) and hepatitis C virus (HCV) were performed to ensure no abnormalities warranting exclusion. LTBI was excluded at screening by T-SPOT.TB (Oxford Immunotec, UK) or QuantiFERON^®^-TB Gold In-Tube test.

PBMC and serum samples from human Study 2 were collected from a previously-described cohort of adult male military recruits who had recently arrived in the UK from Nepal (45). Volunteers shown to be LTBI-negative by T-SPOT.TB ELISpot assay (Oxford Immunotec, UK) and without known history or physical evidence of prior BCG vaccination were enrolled into the current study (group A, n=11), together with a small group of historically BCG-vaccinated individuals who received no intervention (group B, n=4). Ethical approval was granted by the Ministry of Defence Research Ethics Committee (MODREC 237/PPE/11), and all participants provided written informed consent. Group A received a single ID vaccination of BCG SSI at a standard dose (2-8 x 10^5^ CFU) in accordance with current British Army public health policy, and samples were collected at baseline and follow-up visits at 7 days and 70 days.

#### 2.1.2 Macaque Samples

Macaque serum and/or plasma samples used in the ELISA studies described here were collected as part of five independent historical studies of BCG vaccination at Public Health England (PHE), further details of which are provided in **Table 1**. Animals were randomized by socially-compatible group to receive an adult human dose of BCG Danish strain 1331 (SSI, Copenhagen) 2-8x10^5^ CFU ID into the upper arm under sedation, or to be unvaccinated controls. The BCG vaccine was prepared and administered according to the manufacturer’s instructions for preparation of vaccine for administration to human adults, by addition of 1 ml Sauton’s diluent to a lyophilized vial. In all cases, animals were obtained from established breeding colonies at PHE in the UK, which were captive-bred for research purposes, and a single animal was considered an experimental unit. They were provided with enrichment in the form of food and non-food items on a daily basis; animal welfare was monitored daily. Study design and procedures were approved by the Public Health England Animal Welfare and Ethical Review Body and authorized under an appropriate UK Home Office project license. Animals were housed in compatible social groups and in accordance with the Home Office (UK) Code of Practice for the Housing and Care of Animals Used in Scientific Procedures (1989) and the National Centre for Refinement, Reduction and Replacement (NC3Rs) Guidelines on Primate Accommodation, Care and Use, August 2006 (NC3Rs, 2006).

**Table 1:** Summary of macaque samples used in antibody studies.

Study	Species	Genotype	Age (yrs)	Sex	Groups (n)	Times	Ref.
**1**	Rhesus	Indian	14-15	14 F	Naïve controls (5)	D0	n/a
				1 M	BCG vaccinated (10)	D28	
						D56	
**2**	Rhesus	Indian	2.9-3.2	M	Naïve controls (4)	D0	(46)
					BCG vaccinated (5)	D56	
**3**	Rhesus	Indian	3	M	BCG vaccinated (6)	D0	(15)
						D56	
						D140	
**4**	Rhesus	Indian	3.8-4.2	M	BCG vaccinated (6)	D0	(47)
						D56	
						D140	
**5**	Cynomolgus	Mauritian	2.3-3.7	M	BCG vaccinated (12)	D0	n/a
						D56	
						D140	

### 2.2 Enzyme-Linked Immunosorbant Assay

ELISAs were performed as previously described (48). The antigens used were PPD from *M.tb* (Statens Serum Institut (SSI), Denmark) at a concentration of 5 µg/ml; whole BCG SSI at a concentration of 5×10^5^ CFU/ml, *M.tb* H37Rv cell membrane fraction, culture filtrate or whole cell lysate each at a concentration of 2 µg/ml; or lipoarabinomannan (LAM) at a concentration of 1 µg/ml (BEI resources repository). Plates were coated with 50 µl of antigen and incubated at 4°C overnight. Samples were prepared by diluting test serum and positive/negative control serum 1:10, (human Study 1 and macaque studies) or 1:100 (human Study 2), and 50 µl was added per well for 2 hours. For human samples, secondary antibody [goat anti-human IgG (γ-chain-specific), goat anti-human IgA (α-chain-specific), or goat anti-human IgM (µ-chain-specific) alkaline phosphatase conjugate, Sigma Aldrich, MO, US] was diluted 1:1000 and 50 µl was added per well for 1 hour. For macaque samples, secondary antibody [goat anti-monkey IgG (γ-chain-specific), goat anti-monkey IgA (α-chain-specific), or goat anti-monkey IgM (µ-chain-specific) alkaline phosphatase conjugate, Rockland Immunochemicals, PA, US]. was diluted 1:750 and 50 µl added per well for 1 hour. 50 µl of p-nitrophenyl phosphate (pNPP) development buffer was added to each well and the plates were read every 10 minutes using a Model 550 Microplate Reader (Bio-Rad, UK) until the positive control reached a predetermined OD_405_ that was consistent across plates. Reported values represent the mean OD_405_ values of triplicate negative controls subtracted from the mean OD_405_ of triplicate samples.

### 2.3 *Ex Vivo* B Cell Enzyme-Linked Immunospot Assays

#### 2.3.1 Antibody-Secreting Cell ELISpot

ELISpot plates were coated in triplicate with 50 µl/well of BCG SSI at 4x10^6^ CFU/ml. Sterile phosphate-buffered saline (PBS) was used for negative controls. For the detection of total IgG-secreting cells, six wells were coated with 50 µl/well of polyvalent goat anti-human IgG at 50 µg/ml in PBS. Plates were incubated overnight at 4°C and then washed three times with sterile PBS and blocked with R10 media (100 µl/well) for 1 hour at 37°C. Cryopreserved PBMCs were thawed and prepared as previously described (45) and resuspended to a concentration of 5x10^6^ cells/ml in R10. 50 µl of cell suspension was added to the negative control wells and to the first three antigen-coated wells per volunteer. Cells were diluted 1:2 in R10 for the second three antigen-coated wells per volunteer. PBMC dilutions for the total IgG controls were 1:1, 1:25 and 1:50 in R10; 50 µl of the IgG control dilutions were added to duplicate wells. Plates were incubated at 37°C, 5% CO_2_ for 16-18 hours. Cells were then discarded and plates washed six times with PBS-Tween. 50 µl/well of anti-human γ-chain specific IgG conjugated to ALP and diluted 1:5000 in PBS was added and plates incubated for 4 hours at room temperature. Plates were then washed 6 times with PBS-Tween. 50 µl/well of developer was added for 3-5 minutes until spots developed. Plates were washed thoroughly and dried overnight before counting on an AID ELISpot reader. Frequencies of antigen-specific ASCs were calculated by subtracting the mean count of the negative control wells from the test antigen wells and correcting for the number of PBMC in the well. ASC frequency was reported as both the number of ASCs per 1x10^6^ PBMC and the percentage of total IgG-secreting cells. Responses were considered positive if the count was two or more spots in each replicate well, and the total number of spots in the antigen-coated wells twice that observed in the blank control wells.

#### 2.3.2 Memory B Cell ELISpot

For each volunteer, 500 µl of thawed PBMC at a concentration of 2x10^6^ cells/ml in R10 media was added to six wells of a 24-well flat-bottom tissue culture plate. 500 µl of Mitogen stimulation mix containing *Staphylococcus aureus* Cowan (1:2400 dilution), CpG (5 µg/ml) and pokeweed mitogen (1:6000 dilution from a 1-mg/ml stock), was added to five wells per volunteer. 500 µl R10 was added to an unstimulated well as a control. Plates were incubated at 37°C, 5% CO_2_, for 6 days. On day 5, ELISpot plates were coated overnight as described above. On day 6, plates were washed and blocked as described. The cultured cells set up on day 0 for each volunteer were harvested by gentle resuspension and the stimulated cells and unstimulated cells were each pooled, washed twice by centrifugation at 700 g for 5 minutes at room temperature, resuspended in R10 and counted. Cells were then resuspended at 2x10^6^ cells/ml in R10, and 100 µl was added to the negative control wells and the first three antigen-coated wells. For the total IgG wells, dilutions of 1:1, 1:50 and 1:100 were added to each well in duplicate. 100 µl of unstimulated cells were added to duplicate IgG wells. Plates were then incubated at 37°C, 5% CO_2_, for 16-18 hours and developed and counted as described above. Frequencies of mBC were shown as the number of BCG-specific mBC-derived ASC per million cultured PBMC and as the proportion of mBC-derived ASC of total IgG-secreting ASC.

### 2.4 Functional Assays

#### 2.4.1 Direct PBMC Mycobacterial Growth Inhibition Assay

MGIAs were performed as previously described (48), co-culturing 3×10^6^ PBMC and ~500 CFU BCG Pasteur Aeras in a volume of 480 μl RPMI (containing 2 mM l-glutamine and 25 mM HEPES), plus 120 μl autologous serum per well of a 48-well-plate for 96 hours at 37°C and 5% CO_2_. At the end of the culture period, co-cultures were added to 2 ml screw-cap tubes and centrifuged at 15,300 g for 10 minutes. During this time, 500 µl sterile water was added to each well to lyse adherent monocytes. Supernatants were removed from the 2 ml screw-cap tubes, and water from the corresponding well added to the pellet. Tubes were pulse vortexed and the full volume of lysate transferred to BACTEC MGIT tubes supplemented with Polymyxin-B, Amphotericin-B, Nalidixic acid, Trimethoprim, Azilocillin (PANTA) antibiotics and Oleic Albumin Dextrose Catalase (OADC) enrichment broth (Becton Dickinson, UK). Tubes were placed on the BACTEC 960 instrument (Becton Dickinson, UK) and incubated at 37°C until the detection of positivity by fluorescence. On day 0, duplicate direct-to-MGIT viability control tubes were set up by inoculating supplemented BACTEC MGIT tubes with the same volume of mycobacteria as the samples. The time to positivity (TTP) read-out was converted to log_10_ CFU using stock standard curves of TTP against inoculum volume and CFU. Results are presented as normalized BCG growth (log_10_ CFU of sample - log_10_ CFU of growth control).

#### 2.4.2 IgG Purification by Protein G Affinity Chromatography and Complement Depletion

IgG fractions from 300 μl plasma from each of 9 volunteers from human Study 1 (selected according to those with a sufficient volume available) were purified by affinity chromatography on HiTrap Protein G HP columns according to manufacturer’s instructions (GE-Healthcare). The IgG fraction was eluted with 430 μl of 200 mM Glycin buffer (pH 2.0; 0.2 mL/min flow) and neutralized at pH 7.0 with 1 M Tris-HCl buffer pH 11.0. To confirm complement depletion in purified IgG fractions, MILLIPLEX MAP Human Complement Magnetic Bead Panel 1 and 2 kits (Merck Millipore, USA) were used according to manufacturer’s instructions. Complement protein levels in 200x diluted samples were quantified by 5PL logistic regression algorithms built into the Bioplex Manager 6 software using reference standards, and measurements were run on a Bioplex-200 instrument (BIO-RAD, Hertfordshire, UK).

#### 2.4.3 Preparation of BCG-GFP

BCG Montreal (ATCC 35735) containing pEGFP cloned under the control of mycobacterial 19 kDa promoter was kindly provided by Dr Rajko Reljic from St George’s University of London, whereinafter designated as BCG-GFP. Cryopreserved BCG-GFP was thawed and grown in medium containing Middlebrook’s 7H9 broth (BD Biosciences, UK) supplemented with 10% OADC enrichment (BD Biosciences, UK), 0.2% glycerol and 0.05% tyloxapol at 37°C, in aerobic conditions, on a shaker at 200 rpm until it reached log phase and an OD of 1.0 by spectrophotometry, which is equivalent to ~1x10^7^ CFU.

#### 2.4.4 Antibody-Dependent Cellular Phagocytosis

1x10^6^ THP-1 monocytes (ATCC TIB-202) per well in 6-well plates were induced into phagocytic macrophage-like cells by stimulation with 200 nM Phorbol 12-Myristate Acetate (PMA) (Sigma-Aldrich, UK) for 48 hours followed by a resting period without PMA of 24 hours. Prior to infection, BCG-GFP was incubated for 1 hour at room temperature in 2 ml of RPMI 1640 with either diluted serum (1:100) or a final concentration of purified total IgG of 50 μg, 100 μg or 500 μg. Triplicate negative control wells were included in each experiment that contained neither serum nor purified IgG. In parallel, THP-1 derived macrophages were pre-incubated for 1 hour with a final concentration of 100 μg/ml of Purified NA/LE Human Fc Block (BD Biosciences, UK). For *in vitro* infection, adherent cells were infected with BCG-GFP at a multiplicity of infection (MOI) of 5:1 (CFU:cell) and incubated for 4 hours. Macrophages were washed thrice with cold sterile PBS to remove extracellular BCG-GFP and incubated for 15 minutes with Accutase cell detachment solution (BD Biosciences, UK) before final recovery with sterile cell scrapers. Before fixation, cells were stained with a Live-or-Dye fixable viability stain (V450 fluorochrome, Biotium) and washed with PBS. Cells were then fixed with 4% paraformaldehyde for 20 minutes, followed by staining for 30 minutes with anti-CD11b antibody (APCy7 fluorochrome, BD Biosciences, UK). BCG-GFP+ total live cells were quantified by flow cytometry. Samples were run on an LSR II flow cytometer and the data analyzed using FlowJo version 10.8 (TreeStar Inc, Ashland, USA). Each sample was run in duplicate and the results are representative of three experiments.

#### 2.4.5 Opsonization

After harvesting BCG-GFP by centrifugation at 3000 g for 15 minutes and washing twice with PBS, bacterial clumps were dissociated by sonication for 10 minutes and dispersion through a 27G needle. A single cell suspension of 5x10^6^ CFU BCG-GFP was incubated for 2 hours at room temperature in 2 ml of sterile PBS with either diluted plasma (1:10, 1:100 or 1:1000 dilution) or 50 μg, 100 μg or 500 μg of purified total IgG. BCG was washed twice by centrifuging at 4000 g for 10 minutes and re-suspending in sterile PBS, labeled with anti-human IgG Fc antibody (V450 fluorochrome, BD Biosciences, UK) for 30 minutes, and then fixed with 4% paraformaldehyde for 20 minutes. Percentage opsonization was defined as the percentage of IgG+ BCG-GFP by flow cytometry gated by GFP+ or by FSC/SSC for total BCG, adapted from Barr et al. (49). Relative MFI per cell was defined as the median fluorescence intensity divided by the number of anti-IgG+ events detected.

### 2.5 Statistical Analysis

**Figure 1:**
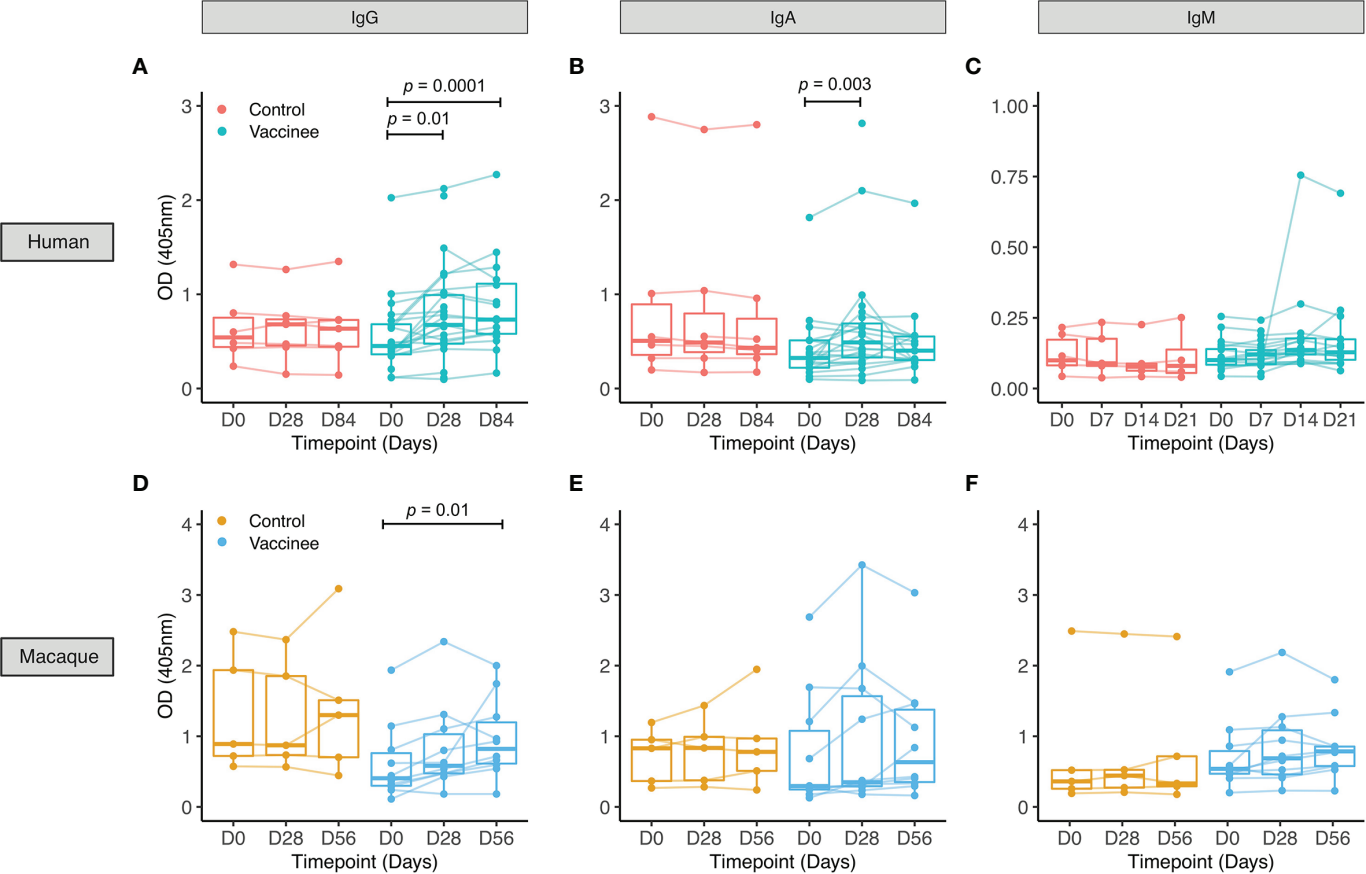
PPD-specific antibody responses to BCG vaccination in healthy UK adults and rhesus macaques. Serum was collected from volunteers enrolled into human Study 1 who were either unvaccinated controls (red) or received BCG vaccination (cyan) **(A–C)**, and from rhesus macaques enrolled into macaque Study 1 which were either unvaccinated controls (yellow) or received BCG vaccination (blue) **(D–F)**. PPD-specific IgG **(A, D)**, IgA **(B, E)** and IgM **(C, F)** responses were measured over time. Points represent the mean of triplicate values, boxes indicate the median value with the interquartile range (IQR) and the upper whisker extends to the largest value no further than 1.5 * IQR from the hinge, the lower whisker extends from the hinge to the smallest value at most 1.5 * IQR from the hinge. Outliers are plotted individually. A mixed effects analysis with Dunnett’s correction for multiple comparisons was conducted on logged values **(A–C)** or a Friedman test with Dunn’s correction for multiple comparisons **(D–F)** was performed to compare the BCG vaccine-induced response between post-vaccination and baseline time-points.

Data were analyzed using GraphPad Prism v.9.2 and SPSS v.27. Normality was determined by Shapiro-Wilk. Longitudinal data was analyzed using a one-way ANOVA with Dunnett’s correction for multiple comparisons (all time-points vs. baseline) for normally-distributed data or a Friedman test with Dunn’s correction for multiple comparisons (all time-points vs. baseline) for non-normally distributed data. For studies with random missing values due to sample unavailability (**Figure 1**), data was logged and analyzed using a mixed-effects model with Dunnett’s correction for multiple comparisons (all time-points vs. baseline). When comparing two conditions (eg. serum vs. plasma), a paired t-test (for normally-distributed data) or Wilcoxon signed-rank test (for non-normally distributed data) was conducted. Associations were determined using a Spearman’s rank correlation.

## 3 Results

### 3.1 BCG Vaccination in Humans and Macaques Induces Higher Levels of PPD-Specific Antibodies Compared With Baseline

For human Study 1 in UK adults, there was a similar age range of volunteers recruited to each group (mean 27 years, range 18-42 for BCG vaccinees; mean 30 years, range 20-45 for controls). 74% of vaccinees and 50% of controls were female. Using serum from this study, we observed a significant increase in PPD-specific IgG at 28 and 84 days and IgA at 28 days post-BCG vaccination compared with baseline, but no significant change in IgM (**Figures 1A**–**C**). There were no differences in antibody responses between males and females at any time-point or in fold change following BCG vaccination for any of the three isotypes (data not shown).

In rhesus macaques enrolled into macaque Study 1, we observed a significant increase in PPD-specific IgG in serum at 56 days post-BCG vaccination compared with baseline, but no significant change in IgA or IgM (**Figures 1D**–**F**). Using serum collected from rhesus macaques (macaque Studies 3 and 4) and cynomologus macaques (macaque Study 5), we compared responses between the two species at the same time-points following BCG vaccination with the standard dose. We observed similarly significant induction of PPD-specific IgG at 56 and 140 days post-BCG vaccination in the two species (**Figure S1A**). There was no difference in fold change in IgG response following BCG vaccination between the species at either time-point (**Figure S1B**).

No differences were observed over time in the control group for any of the isotypes measured for either humans or macaques. There was no difference in fold change in PPD-specific IgG or IgA levels at 28 days following BCG vaccination (the only directly comparable time-point) between humans and macaques (**Figure S2**). We observed several associations between fold change following BCG vaccination in levels of different PPD-specific isotypes in both humans and macaques, as summarized in **Tables S1**–**S4**.

Similar results were obtained for IgG, IgA and IgM responses in plasma in both humans and macaques (**Figure S3**). PPD-specific IgG, IgA and IgM levels were significantly higher in serum compared with plasma at all time-points in humans and some time-points in macaques (**Figure S4**). There was no difference in fold change following BCG vaccination between serum and plasma for any of the isotypes at any of the time-points measured in either species (**Figure S5**). The correlation between PPD-specific antibodies in the BCG vaccinated group in serum and plasma was strong at all time-points for all isotypes in both species (**Figure S6**).

### 3.2 Association Between Levels of Serum Antibodies Specific to Different Mycobacterial Fractions

**Figure 2:**
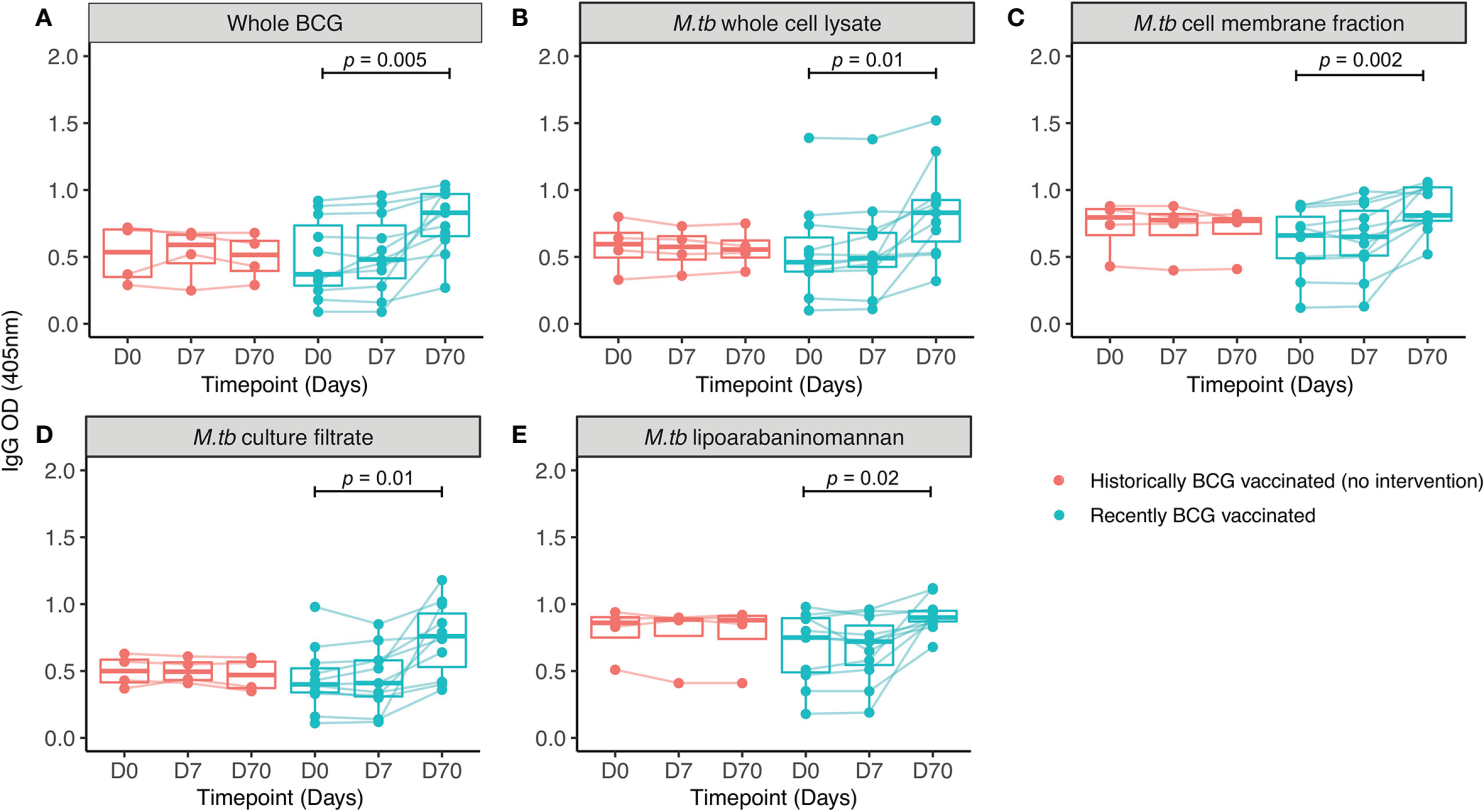
IgG responses to different mycobacterial fractions in serum collected from Nepalese military recruits. Serum samples were taken from Nepalese adults enrolled into human Study 2 (red = historically BCG-vaccinated individuals who received no intervention, cyan = BCG-naïve individuals who received BCG vaccination). IgG responses to whole BCG **(A)**, *M.tb* whole cell lysate **(B)**, *M.tb* cell membrane fraction **(C)**, *M.tb* culture filtrate **(D)** or *M.tb* lipoarabaninomannan **(E)** were measured. Points represent the mean of triplicate values, boxes indicate the median value with the interquartile range (IQR) and the upper whisker extends to the largest value no further than 1.5 * IQR from the hinge, the lower whisker extends from the hinge to the smallest value at most 1.5 * IQR from the hinge. Outliers are plotted individually. A repeated measures one-way ANOVA with Dunnett’s multiple comparisons test was used to compare between time-points in the BCG vaccinated group.

In human Study 2, all volunteers were male Nepalese military recruits with a similar median age in groups A and B (18.9 vs. 20.5 years respectively). IgG responses specific to multiple mycobacterial fractions were measured at baseline, 7 and 70 days post-BCG vaccination in serum. At 70 days, there was a significant increase in IgG specific for whole BCG, whole-cell lysate, cell membrane fraction, culture filtrate and lipoarabinomannan (LAM) compared with baseline (**Figures 2A**–**E**). There was no change over time in the level of IgG specific to any of the antigens in the historically BCG-vaccinated group who received no intervention. The greatest BCG-induced fold change was in IgG specific for *M.tb* whole cell lysate and *M.tb* culture filtrate (median fold change = 1.7 and 1.6 respectively), while the smallest was against *M.tb* cell membrane fraction and LAM (median fold change = 1.2 and 1.2). However, there was a significant association in BCG-induced fold change in IgG between all mycobacterial fractions (**Table 2**). Responses to *M.tb* cell membrane fraction and LAM in the historically BCG vaccinated group were similar in magnitude to day 70 responses in the recently BCG vaccinated group, which may reflect a more sustained response to these fractions (**Figures 2C, E**). There was no difference in fold change in the BCG-specific IgG response following BCG vaccination between individuals in human Study 1 (from a non-TB endemic country) at 84 days and individuals from human Study 2 (from a TB-endemic country) at 70 days (data not shown).

**Table 2:** Spearman’s correlations between fold change (post-vaccination/baseline) in IgG specific to different mycobacterial fractions following BCG vaccination in serum collected from Nepalese military recruits.

	Whole BCG	*M.tb* Whole cell lysate	*M.tb* cell membrane fraction	*M.tb* culture filtrate	LAM
Whole BCG		.955	.791	.909	.809
	X	.000	.004	.000	.003
		***	**	***	**
*M.tb* whole cell lysate	.955		.745	.964	.718
	.000	X	.008	.000	.013
	***		**	***	*
*M.tb* cell membrane fraction	.791	.745		.782	.927
	.004	.008	X	.004	.000
	**	**		**	***
*M.tb* culture filtrate	.909	.964	.782		.736
	.000	.000	.004	X	.010
	***	***	**		**
LAM	.809	.718	.927	.736	
	.003	.013	.000	.010	X
	**	*	***	**	

* indicates a p-value of <0.05, ** indicates a p-value of <0.01, and *** indicates a p-value of <0.001.

IgG levels specific to PPD and whole BCG were compared in serum from human Study 1. There was a significant increase in IgG specific to whole BCG and PPD at 28 days post-BCG vaccination (**Figures S7A, B**), and a significant correlation between IgG specific to the two antigens following vaccination (**Figure S7C**). Similarly, in macaque Study 2 there was a significant increase in both BCG-specific and PPD-specific IgG at 56 days following BCG vaccination (**Figures S7D, E**) with a significant correlation between the two measures (**Figure S7F**). There was no change in IgG levels in the control group over time in either study. Finally, antibody responses to *M.tb* whole cell lysate (MTB WCL) were measured in serum collected from rhesus macaques enrolled into macaque Study 1. There was an increase in MTB WCL-specific IgG and IgA but not IgM at days 28 and 56 post-BCG vaccination (**Figures S8A**–**C**), and we observed significant associations between PPD-specific and MTB WCL-specific IgA and IgM at day 28, and IgG at day 56 (**Figures S8D**–**F**).

### 3.3 BCG-Specific IgG-Secreting Plasmablasts and Memory B Cells Are Induced Following BCG Vaccination

**Figure 3:**
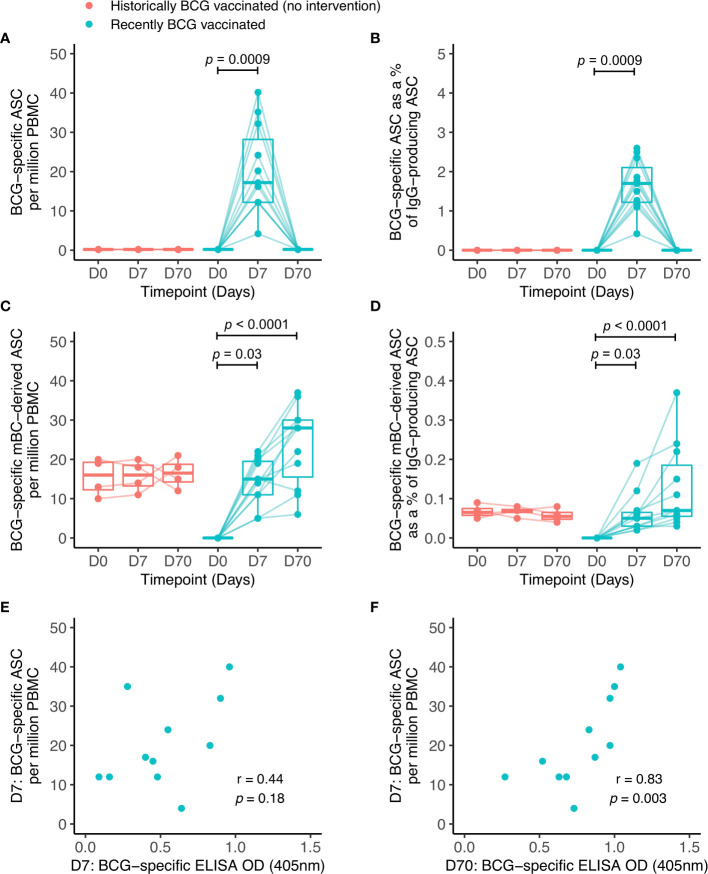
BCG-specific IgG-secreting plasmablast (ASC) and memory B cell-derived ASC responses following BCG vaccination. ASC and mBC-derived ASC responses were determined at baseline, 7 days and 70 days using cells collected from Nepalese military recruits enrolled into human Study 2 (red = historically BCG-vaccinated individuals who received no intervention, cyan = BCG-naïve individuals who received BCG vaccination). ASC responses are presented as the number of BCG-specific ASCs per million PBMC **(A)** and as the proportion (%) of total IgG-secreting ASC **(B)**. mBC responses are shown as the number of BCG-specific mBC-derived ASC per million cultured PBMC **(C)** and as the proportion (%) of mBC-derived ASC of total IgG-secreting ASC **(D)**. Points represent the mean of triplicate values, boxes indicate the median value with the interquartile range (IQR) and the upper whisker extends to the largest value no further than 1.5 * IQR from the hinge, the lower whisker extends from the hinge to the smallest value at most 1.5 * IQR from the hinge. Outliers are plotted individually. A Friedman test with Dunn’s multiple comparisons test was used **(A–D)**. A Spearman rank correlation was performed between ASC responses and BCG-specific IgG responses measured at 7 days **(E)** and 70 days **(F)** after BCG vaccination.

To investigate the origin of the antibodies observed, B cell ELISpot assays were performed to detect BCG-specific IgG-secreting cells indicative of either plasmablasts measured following an overnight assay, or mBCs enumerated following several days of culture and stimulation with polyclonal mitogens. The antigen used was the same BCG strain given during vaccination (BCG-SSI). The transient induction of plasmablasts was noted at 7 days following BCG vaccination in Group A (mean of 20 ASC per million PBMC or 1.7% of total IgG+ ASC) which was undetectable by day 70 (**Figures 3A, B**). No plasmablast responses were seen in Group B (historically BCG vaccinated but receiving no intervention). There was an increase in the memory B cell response at 7 and 70 days post-BCG vaccination in Group A, and Group B showed persisting stable BCG-specific mBC responses (mean of 16 mBC-derived ASC per million cultured PBMC or 0.06% of IgG+ mBC-derived ASC) (**Figures 3C, D**), reflecting their historical BCG vaccination status. There was a trend towards a correlation between ASC responses at 7 days and BCG-specific IgG levels at 7 days (**Figure 3E**), which was significant at 70 days (**Figure 3F**). There were no associations between mBC-derived ASC responses and BCG-specific IgG levels at either time-point studied.

### 3.4 BCG Vaccine-Induced Antibodies Play a Functional Role *In Vitro*

A workflow for the functional assays performed is provided in **Figure S9**. The direct PBMC MGIA was conducted using cryopreserved cells and autologous serum collected at baseline and 84 days post-BCG vaccination from a subset of volunteers, n=9, enrolled into human Study 1. To identify a vaccine-induced response, we compared control of mycobacterial growth mediated by baseline PBMC combined with baseline serum vs. post-vaccination PBMC combined with post-vaccination serum. To determine whether serum factors such as antibodies were contributing to the observed effect, we then swapped serum by time-point. Therefore post-vaccination serum was combined with baseline PBMC and baseline serum was combined with post-vaccination PBMC. We hypothesized that if serum factors were contributing to mycobacterial growth inhibition as well as cellular immunity, the vaccine response in this assay would be diminished or lost when serum was swapped by time-point.

**Figure 4:**
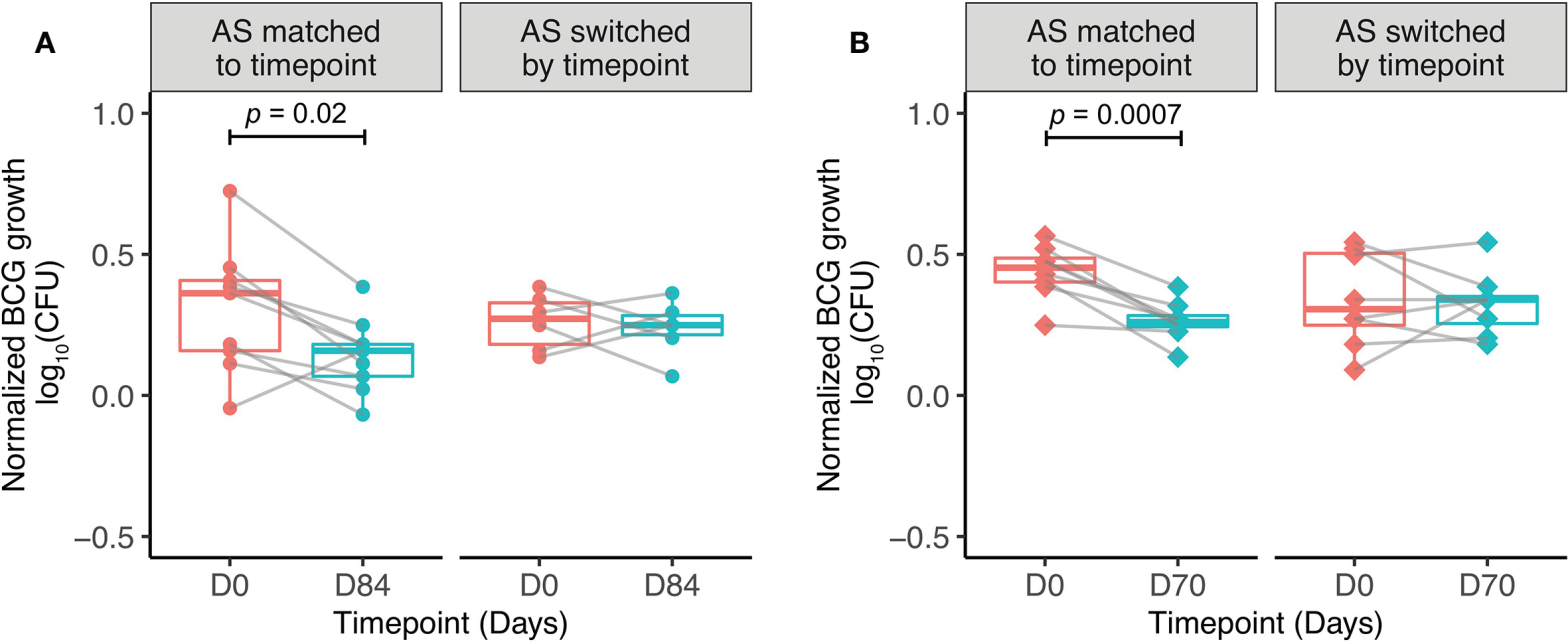
Serum factors contribute to BCG-induced control of mycobacterial growth *in vitro*. Samples were used from n=9 healthy UK adults enrolled into human Study 1 **(A)** and n=9 healthy Nepalese military recruits enrolled into human Study 2 **(B)**, all of whom received ID BCG vaccination. The direct MGIA was performed using PBMC collected at baseline and 84 days **(A)** or 70 days **(B)** post-BCG vaccination, co-cultured with BCG and either autologous serum matched to time-point, or autologous serum switched by time-point ie. post-vaccination serum cultured with baseline PBMC and baseline serum cultured with post-vaccination PBMC. Normalized BCG growth = (log_10_ CFU of sample - log_10_ CFU of growth control). Points represent the mean of duplicate values, boxes indicate the median value with the interquartile range (IQR) and the upper whisker extends to the largest value no further than 1.5 * IQR from the hinge, the lower whisker extends from the hinge to the smallest value at most 1.5 * IQR from the hinge. Outliers are plotted individually. A paired t-test was used to compare between time-points.

When serum was matched to time-point, there was a significant improvement in control of mycobacterial growth at 84 days post-BCG vaccination compared with baseline, as previously reported (50) (**Figure 4A**). When serum was swapped between pre- and post-vaccination time-points (baseline PBMC cultured with day 84 serum, and day 84 PBMC cultured with baseline serum), control of mycobacterial growth was no longer different between time-points. This finding was validated using samples (n=9) collected at baseline and 70 days from Study 2. When serum was matched to time-point, there was a significant improvement in control of mycobacterial growth at 70 days post-BCG vaccination compared with baseline (**Figure 4B**). When serum was exchanged between pre- and post-vaccination time-points, the difference in control of mycobacterial growth was again lost. Control of mycobacterial growth was modestly improved using cells taken at baseline cultured with serum taken at 70 days post-vaccination compared with serum taken at baseline (**Figure 4B**).

**Figure 5:**
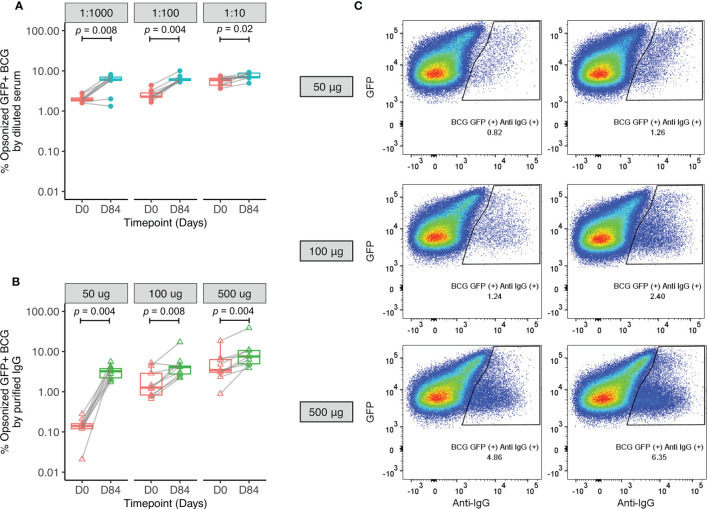
Enhanced opsonization of BCG-GFP with sera or purified IgG following BCG vaccination. Samples were used from n=9 healthy UK adults enrolled into human Study 1. BCG-GFP was incubated with either serum at different dilutions **(A)** or purified IgG at different concentrations **(B)** collected at baseline and 84 days post-BCG vaccination, and the percentage of BCG-GFP that was opsonized was determined by flow cytometry. Points represent the mean of duplicate values, boxes indicate the median value with the interquartile range (IQR) and the upper whisker extends to the largest value no further than 1.5 * IQR from the hinge, the lower whisker extends from the hinge to the smallest value at most 1.5 * IQR from the hinge. Outliers are plotted individually. A Wilcoxon matched-pairs test was used to compare between time-points. Flow plots are shown from a single replicate of one subject at baseline and 84 days post-BCG vaccination using different concentrations of purified IgG **(C)**.

The mechanism by which serum factors may be influencing mycobacterial growth control in the MGIA was explored by measuring opsonization of BCG-GFP and ADCP using THP-1 derived macrophages and diluted serum from human Study 1 (n=9). The flow cytometry gating strategies are shown in **Figure S10**. Opsonization rates were significantly higher when BCG-GFP was incubated with serum from 84 days post-vaccination compared with baseline at all three serum dilutions (**Figure 5A**). To remove the influence of other serum components and differing antibody titers and thus represent a cleaner system, the assay was repeated using standardized amounts of total IgG purified from pre- and post-BCG vaccination plasma. Effective depletion of complement proteins was confirmed by Luminex (**Figure S11**). Opsonization remained significantly increased following vaccination (**Figure 5B, C**). Similar results were obtained when total bacteria (GFP+ and GFP- combined) were gated based on size to account for non GFP-expressing bacteria, and when mean fluorescence intensity (MFI) was used as a read-out to take into account bacteria opsonized with multiple antibodies (**Figure S12**).

**Figure 6:**
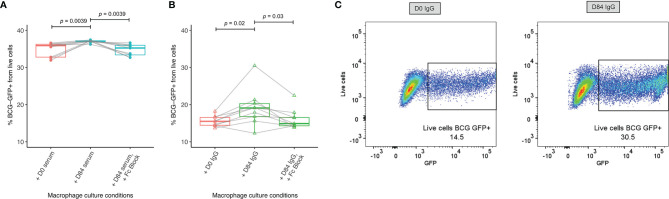
Enhanced macrophage phagocytosis of BCG-GFP with sera or purified IgG following BCG vaccination. Samples were used from n=9 healthy UK adults enrolled into human Study 1. Antibody-dependent cellular phagocytosis was measured using human THP-1 derived macrophages and serum **(A)** or purified IgG **(B)** from baseline and 84 days post-BCG vaccination and the percentage of BCG-GFP that was phagocytosed was determined by flow cytometry. Points represent the mean of duplicate values, boxes indicate the median value with the interquartile range (IQR) and the upper whisker extends to the largest value no further than 1.5 * IQR from the hinge, the lower whisker extends from the hinge to the smallest value at most 1.5 * IQR from the hinge. Outliers are plotted individually. A Wilcoxon matched-pairs test was used to compare between time-points. Flow plots are shown from a single replicate of one subject at baseline and 84 days post-BCG vaccination using purified IgG **(C)**.

Macrophage phagocytosis of BCG-GFP also increased significantly following incubation with post-vaccination sera or purified IgG compared with sera or purified IgG from baseline, and this effect was reversed when cells were first treated with anti-Fc-γ receptor (FcγR) antibody to block binding of the FcγRs (**Figures 6A**–**C**). Following BCG vaccination, there was a correlation between macrophage phagocytosis and opsonization using 500 μg of purified IgG (r=0.7, p=0.04, Spearman’s, data not shown).

## 4 Discussion

We describe the induction of specific IgG following BCG vaccination in two independent human cohorts (one from a TB-endemic and one from a non-endemic region), consistent with several previous reports (51–54). While others observed no change in IgG titer following BCG vaccination, limitations of such studies have included antibody quantification methods, small sample sizes, variable/unspecified time-points and differences in dose and strain of BCG (26, 55–57). Both of our cohorts comprised healthy adults who received a standard dose of BCG SSI and antibodies were measured up to ~3 months post-vaccination. One limitation of our studies is the unequal number of males and females. While it has been suggested that antibody responses to other vaccines may be higher in females (58), we did not observe any such sex differences. Interestingly, the greatest fold change following vaccination was in IgG specific for *M.tb* WCL and culture filtrate, suggesting recognition of secreted as well as surface proteins. The historically BCG vaccinated volunteers showed a sustained humoral response against LAM, and it has previously been reported that anti-LAM or -AM antibodies may contribute to protection from TB (28, 59, 60). AM-specific IgG2, IgA and IgM are also boosted by BCG revaccination (28); antibody responses to BCG revaccination would be interesting to explore further in light of recent efficacy data (17).

While macaques are partially protected by BCG vaccination and considered the most relevant preclinical model for human TB vaccine development, there is a paucity of literature on the antibody response to BCG in this model (61–63). We demonstrate BCG vaccine-mediated induction of specific IgG consistently across five different macaque studies, which included two species and animals of different ages (young adults aged 2.3-4.2 years and older adults aged 14-15 years). Interestingly, IgG and IgA levels were still rising at 56 days post-vaccination; contrary to a recent report that suggested a peak at 28 days (16). Although previous studies have reported that rhesus macaques are more susceptible to acute progressive TB disease and BCG confers lower efficacy compared with cynomolgus macaques of Asian origin (33, 64), the study reported here was in cynomolgus macaques of Mauritian origin which are less genetically diverse with equal susceptibility to TB disease as rhesus macaques (65, 66). We also observed similar fold change in IgG and IgA levels relative to baseline in humans and macaques at the comparable time-point (day 28), offering some confidence in the translatability of this model in this context.

PPD-specific antibody levels in serum and plasma correlated strongly, consistent with the observations of Siev et al. (67), but were modestly yet significantly higher in serum across isotypes. Other studies have similarly noted lower levels of proteins in plasma than serum (68, 69), potentially associated with the addition of anticoagulant to blood prior to obtaining plasma, or the presence of fibrinogen (70). Although this may limit the interchangeability of serum and plasma in the same experiment, fold change in BCG vaccine-induced antibody responses was comparable indicating that sensitivity to detect a vaccine-induced response does not differ. One limitation of this study is that only serum and plasma were available, so we were unable to evaluate secretory IgA which may be of particular relevance against pulmonary infection by blocking mycobacterial entrance and/or modulating proinflammatory responses (71–74). Future work should aim to assess this localized antibody response in addition to those in the systemic blood compartment.

To our knowledge, the specific antibody-secreting B cell response to BCG vaccination has not been previously described. We observed induction of BCG-specific IgG-secreting plasmablasts at 7 days post-BCG vaccination returning to baseline by day 70, consistent with the kinetic of response to other primary vaccinations (40, 41). BCG-specific mBC responses were detectable in the group who had received BCG up to 20 years previously, in concordance with a previous study (43). However, the responses noted here are higher, which may be due to the use of different antigens and different populations. Unfortunately sample availability did not allow the testing of responses to other mycobacterial antigens, but this would be interesting to explore in future – particularly whether the apparently superior durability of LAM-specific IgG responses corresponds to long-lived LAM-specific mBCs. In the previously BCG-naïve group, mBCs induced by BCG vaccination were apparent at 7 days, as per the murine model (75). A study assessing longitudinal mBC responses following exposure to malaria vaccines showed a gradual increase in specific mBCs which peaked at 84 days before declining by 140 days (42). We were unable to define the peak or longevity of mBC responses in our study due to logistical restrictions on collecting later follow-up samples, but one would similarly expect contraction to a persisting residual level over time (76). A further limitation is the lack of sample for flow cytometry-based analysis of antigen-specific cells which would allow definitive identification and quantification of ASCs and mBCs.

Interestingly, we saw relatively high antibody responses at baseline and in the naïve unvaccinated controls across isotypes and studies, which may indicate cross-reactive antibodies induced by environmental exposure to non-tuberculous mycobacteria (77, 78). Others have also noted pre-existing specific antibodies in serum from PPD-negative individuals with no known exposure to *M.tb* (27, 79). It is possible that an element of the ASC response in the Nepalese cohort was due to the generation of plasmablasts from pre-existing mycobacteria-specific mBCs. Indeed, the rapidity and magnitude of the ASC response was comparable to recall responses observed in secondary vaccination studies (40, 42); although this hypothesis is not supported by the lack of mBC responses at baseline in unvaccinated volunteers, they may have been present at frequencies below the limit of detection of the ELISpot assay. It remains to be determined whether baseline reactivity interferes with induction of a humoral response to BCG vaccination in the way it appears to affect IFN-γ responses (80), particularly given the widely-accepted masking/blocking hypotheses for the geographical variation in BCG efficacy (12).

In order to explore a functional role for the antibodies detected, we applied a sum-of-the-parts mycobacterial growth inhibition assay (MGIA) that we have previously optimized as a potential surrogate of TB vaccine-induced protection (48, 50, 81–85). Various immune mechanisms have been proposed to contribute to mycobacterial control in this assay including polyfunctional CD4+ T cells and trained innate immunity (48, 83, 84). In two independent cohorts, we confirmed previous findings of enhanced mycobacterial growth control following BCG vaccination, and further showed that this effect was reduced by exchanging pre- for post-vaccination serum and vice versa. In our second human cohort, we observed enhanced mycobacterial control when baseline cells were cultured with post-vaccination serum compared with baseline cells cultured with baseline serum, although the effect was less pronounced than between baseline cells cultured with baseline serum and post-vaccination cells cultured with post-vaccination serum. This suggests that control of mycobacterial growth in the direct MGIA is mediated by a combination of cellular and humoral immunity, although the mechanism by which antibodies contribute is as yet undetermined.

Our finding of increased opsonization and macrophage phagocytosis of BCG using post-vaccination compared with pre-vaccination serum points to a functional role for BCG vaccine-induced antibodies that may contribute to the mycobacterial growth control observed in the MGIA. This is consistent with other studies reporting enhancement of opsonophagocytosis and mycobacterial growth control *via* antibody-mediated mechanisms (27, 28). That increased opsonization and ADCP following vaccination were also observed using standardized quantities of purified IgG suggests that a) IgG antibodies are mediating this effect at least in part, and b) there may be vaccine-induced qualitative differences in the functional capacity of these antibodies, although the proportion of antigen-specific IgG within total IgG was not standardized. A stronger ADCP effect observed with diluted serum compared to purified IgG suggests that other serum factors such as complement are contributing, although this difference may also be influenced by the low concentrations of purified IgG used. We also provide further evidence that the enhancement of macrophage phagocytosis is FcγR-mediated (28). In future, it would be interesting to adapt the assay for use with primary cells to take into account effects of BCG vaccination on monocytes and how these influence ADCP.

In conclusion, we have demonstrated the BCG vaccine-mediated induction of specific antibodies that are modestly higher in serum than plasma and comparable across humans and macaques in magnitude of vaccine response, as well as serum responses to a range of mycobacterial fractions. We have also shown for the first time the rapid and transient induction of antibody-secreting plasmablasts following BCG vaccination, together with a robust memory B cell response at 70 days post-vaccination. While contracted, a measurable mBC response was still present in volunteers that received BCG vaccination up to 20 years previously, suggesting that this pool is maintained and may contribute to a long-lived humoral response. Finally, we used functional assays to demonstrate a potential contribution of antibodies to BCG vaccine-mediated control of mycobacterial growth that is FcγR-mediated. Future work should aim to perform similar assessments in studies where protection data are available so that it can be determined whether different aspects of the antibody response relate to levels of protection from TB *in vivo*. Further characterization of the BCG-induced response may aid in the design of more efficacious TB vaccine candidates that could benefit from the induction of humoral as well as cellular immunity.

## Data Availability Statement

The raw data supporting the conclusions of this article will be made available by the authors, without undue reservation.

## Ethics Statement

Human Study 1 was approved by the NHS Research Ethics Service (NRES) Committee South Central - Oxford B (REC reference 15/SC/0022), and registered with ClinicalTrials.gov (NCT02380508). All aspects of the study were conducted according to the principles of the Declaration of Helsinki and Good Clinical Practice. Volunteers provided written informed consent prior to screening. Ethical approval for human Study 2 was granted by the Ministry of Defence Research Ethics Committee (MODREC 237/PPE/11). The patients/participants provided their written informed consent to participate in this study. For the macaque studies, study designs and procedures were approved by the Public Health England Animal Welfare and Ethical Review Body and authorized under an appropriate UK Home Office project license.

## Author Contributions

RT, HMcS, MO’S, and MPÁ conceived and designed the work. JB, MO’S, RT, MPÁ, MW, AJ, DW, SSA, SH, SL, SS, SE, AW, and SGS conducted the studies and/or contributed to the acquisition of data. RT, JB, MPÁ, IS, MO’S, and HMcS contributed to the interpretation of the data. RT, JB, and MO’S wrote the paper. All authors contributed to the article and approved the submitted version.

## Funding

This work was funded in part by a small grant awarded to RT from the Royal Society of Tropical Medicine and Hygiene (RSTMH); the European Research Infrastructures for Poverty Related Diseases (EURIPRED), an EC seventh framework program (grant number 312661); TBVAC2020 (grant number 643381); and the Wellcome Trust (HMcS is a Wellcome Trust Investigator, grant code WT 206331/Z/17/Z). Human Study 1 was funded by the Bill & Melinda Gates Foundation (grant number OPP1112389) and human Study 2 was funded by a grant awarded to MO’S from the Wellcome Trust (grant number 103420/Z/13/Z). For the purpose of open access, the author has applied a CC BY public copyright license to any Author Accepted Manuscript version arising from this submission. This work was also supported by the National Institute for Health Research (NIHR) Oxford Biomedical Research Center (BRC).

## Author Disclaimer

The views expressed are those of the author(s) and not necessarily those of the NHS, the NIHR or the Department of Health.

## Conflict of Interest

The authors declare that the research was conducted in the absence of any commercial or financial relationships that could be construed as a potential conflict of interest.

## Publisher’s Note

All claims expressed in this article are solely those of the authors and do not necessarily represent those of their affiliated organizations, or those of the publisher, the editors and the reviewers. Any product that may be evaluated in this article, or claim that may be made by its manufacturer, is not guaranteed or endorsed by the publisher.
