# Supplementary material for: Induction of Functional Specific Antibodies, IgG-Secreting Plasmablasts and Memory B Cells Following BCG Vaccination
Source: Front Immunol. 2022 Jan 5;12:798207. doi: 10.3389/fimmu.2021.798207 (PMC8767055; doi:10.3389/fimmu.2021.798207)
Supplement: Supplementary file 1 [file DataSheet_1.docx]

**Induction of functional specific antibodies, IgG-secreting plasmablasts and memory B cells following BCG vaccination: Supplementary information**

**1.0 Supplementary Figures**


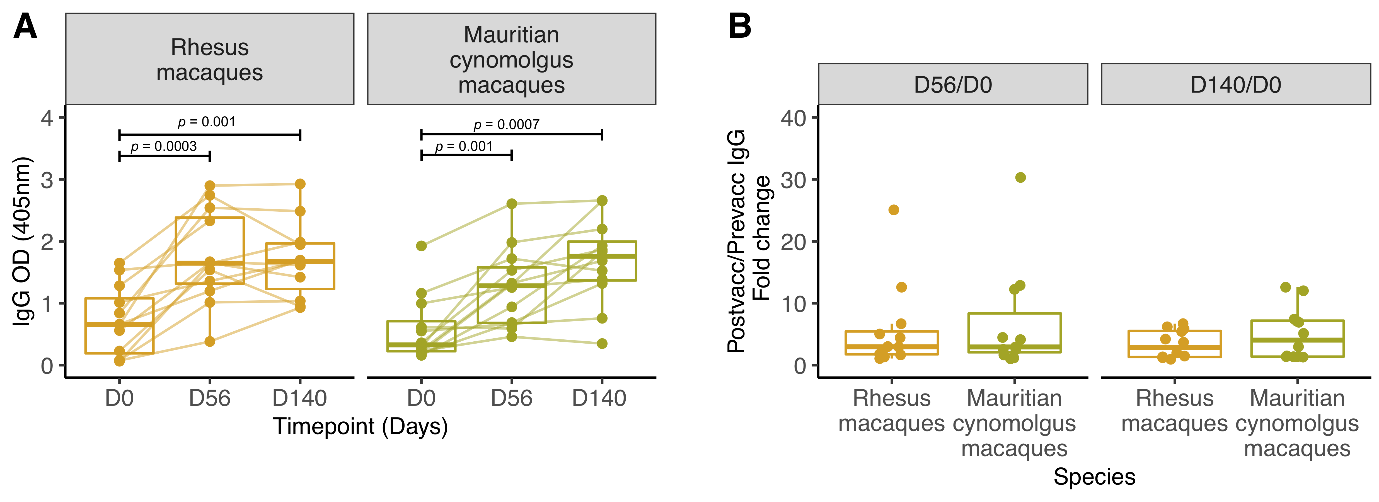
**Figure S1. Comparison of specific IgG responses to BCG vaccination between rhesus and cynomolgus macaques.** PPD-specific IgG levels (**A**) and fold change in IgG (**B**) following BCG vaccination were determined in rhesus macaques (yellow) enrolled in macaque Studies 3 and 4, and Mauritian cynomolgus macaques (green) enrolled in macaque Study 5. Points represent the mean of triplicate values, boxes indicate the median value with the interquartile range (IQR) and the upper whisker extends to the largest value no further than 1.5 * IQR from the hinge, the lower whisker extends from the hinge to the smallest value at most 1.5 * IQR from the hinge. Outliers are plotted individually. For **A**), an RM ANOVA with Dunnett’s multiple comparisons test was performed and for **B**), a Wilcoxon test was performed.


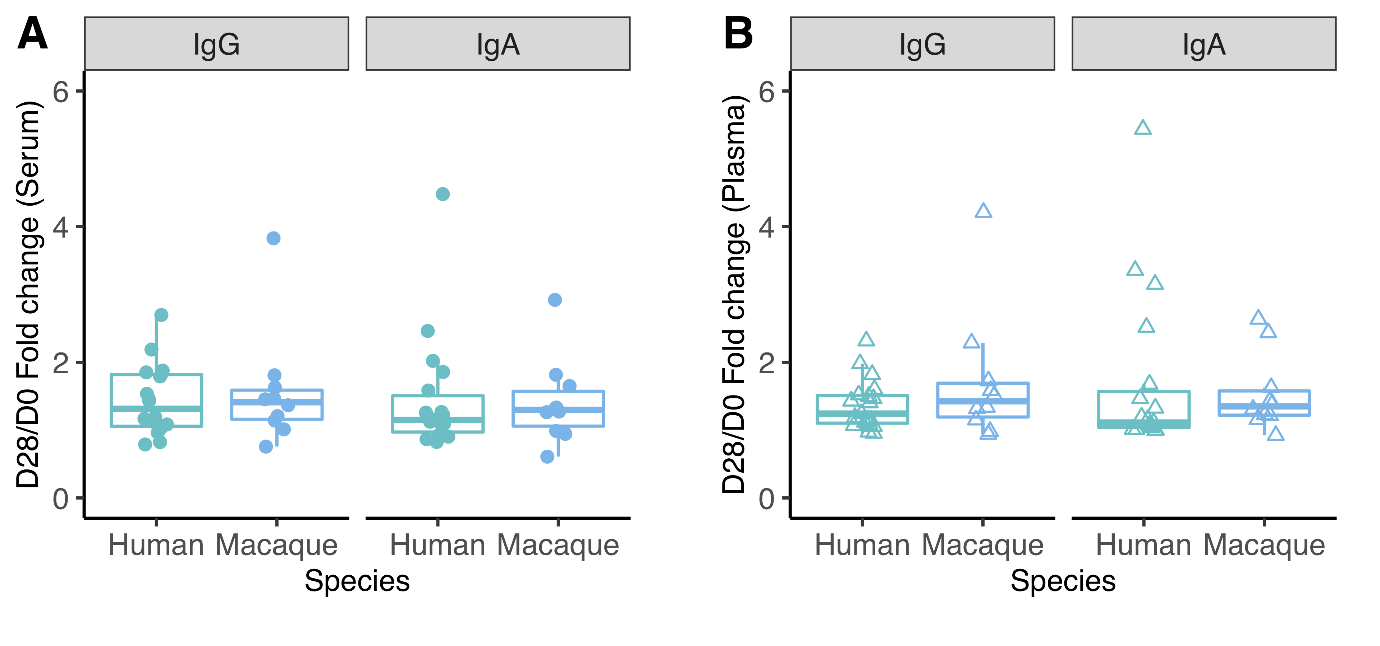
**Figure S2. Comparison of PPD-specific IgG responses to BCG vaccination between humans and macaques.** Fold change (28 days post-BCG vaccination/baseline) in PPD-specific IgG in serum (circles, **A**) and plasma (triangles, **B**) was compared between healthy UK adults enrolled into human Study 1 (cyan) and rhesus macaques enrolled into macaque Study 1 (blue). Points represent the mean of triplicate values, boxes indicate the median value with the interquartile range (IQR) and the upper whisker extends to the largest value no further than 1.5 * IQR from the hinge, the lower whisker extends from the hinge to the smallest value at most 1.5 * IQR from the hinge. Outliers are plotted individually. A Wilcoxon test was performed.


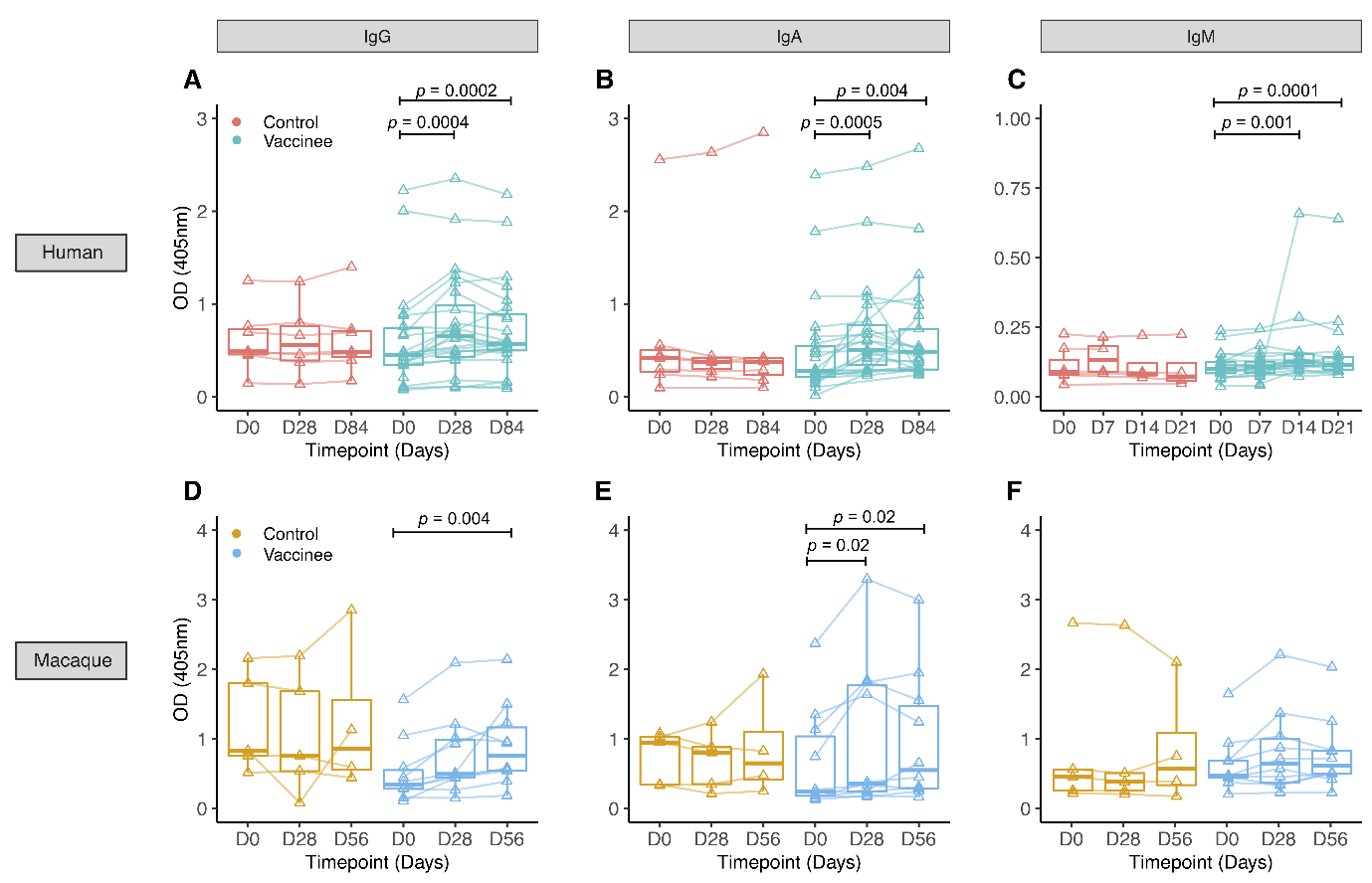


**Figure S3. Plasma PPD-specific antibody responses to BCG vaccination in healthy UK adults and rhesus macaques.** Plasma was collected from volunteers enrolled into human Study 1 who were either unvaccinated controls (red) or received BCG vaccination (cyan) (**A-C**), and from animals enrolled into macaque Study 1 which were either unvaccinated controls (yellow) or received BCG vaccination (blue) (**D-F**). PPD-specific IgG (**A, D)**, IgA (**B, E**) and IgM (**C, F**) responses were measured over time. Points represent the mean of triplicate values, boxes indicate the median value with the interquartile range (IQR) and the upper whisker extends to the largest value no further than 1.5 * IQR from the hinge, the lower whisker extends from the hinge to the smallest value at most 1.5 * IQR from the hinge. Outliers are plotted individually. A mixed effects analysis with Dunnett’s correction for multiple comparisons was conducted on logged values (**A-C**) or a Friedman test with Dunn’s correction for multiple comparisons (**D-F**) was performed to compare the BCG vaccine-induced response between post-vaccination and baseline time-points.


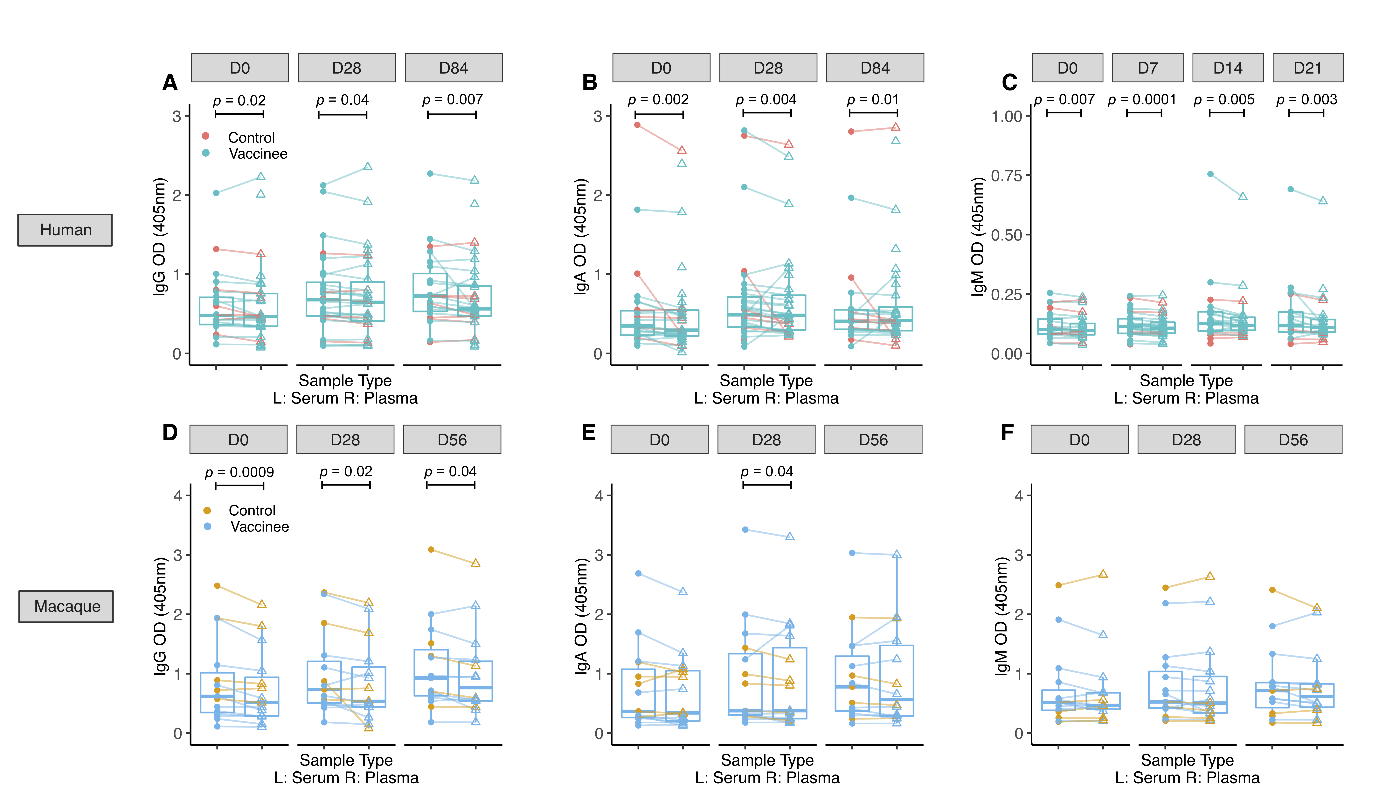


**Figure S4. Comparison of PPD-specific antibody levels between serum and plasma.** PPD-specific IgG (**A**, **D**), IgA (**B**, **E**) and IgM (**C**, **F**) responses were compared between serum (circles) and plasma (triangles) collected from healthy UK adults enrolled into human Study 1 (**A-C**) and rhesus macaques enrolled into macaque Study 1 (**D-F**). Points represent the mean of triplicate values, boxes indicate the median value with the interquartile range (IQR) and the upper whisker extends to the largest value no further than 1.5 * IQR from the hinge, the lower whisker extends from the hinge to the smallest value at most 1.5 * IQR from the hinge. Outliers are plotted individually. Wilcoxon tests were performed.


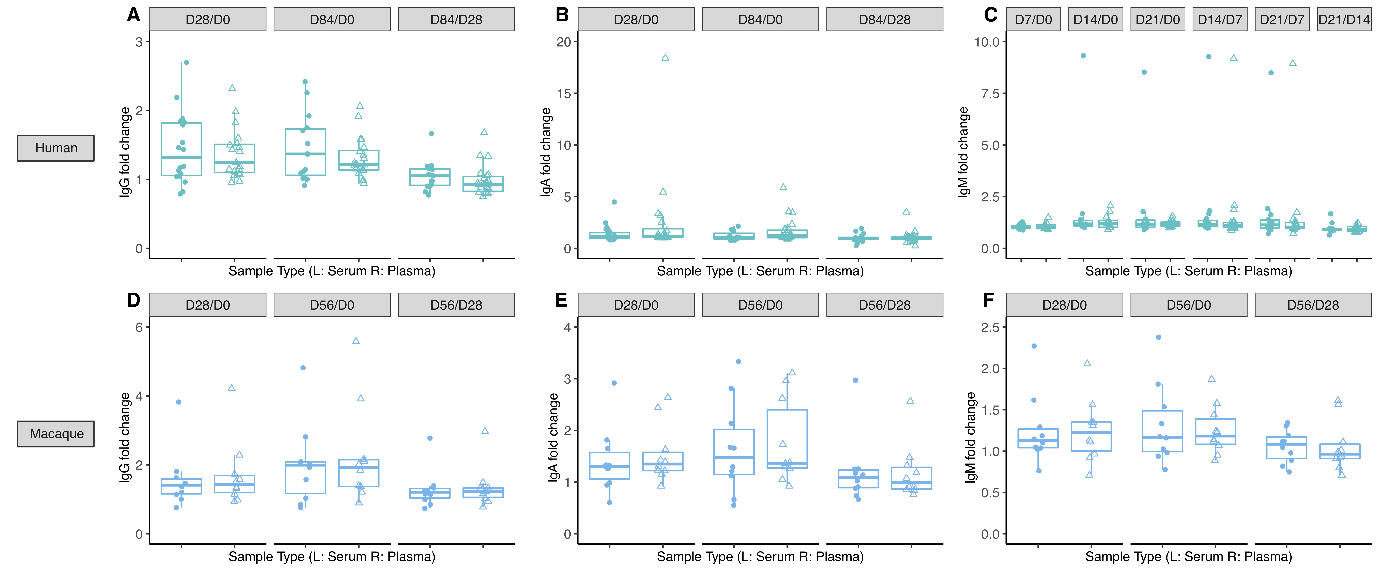


**Figure S5. Comparison of fold change in PPD-specific antibody levels following BCG vaccination between serum and plasma.** Fold change in PPD-specific antibody responses following BCG vaccination was compared between serum (circles) and plasma (triangles) collected from healthy UK adults enrolled into human Study 1 (cyan, **A-C**) and rhesus macaques enrolled into macaque Study 1 (blue, **D-F**). Fold change in IgG (**A**) and IgA (**B**) responses were compared between baseline and 28 days post-vaccination, baseline and 84 days post-vaccination, and 84 and 28 days post-vaccination in humans. Fold change in IgM responses (**C**) were compared between baseline and 7 days post-vaccination, baseline and 14 days post-vaccination, baseline and 21 days post-vaccination, 14 and 7 days post-vaccination, 21 and 7 days post-vaccination, and 21 and 14 days post-vaccination. Fold change in IgG (**D**), IgA (**E**) and IgM (**F**) responses were compared between baseline and 28 days post-vaccination, baseline and 56 days post-vaccination, and 56 and 28 days post-vaccination in macaques. Points represent the mean of triplicate values, boxes indicate the median value with the interquartile range (IQR) and the upper whisker extends to the largest value no further than 1.5 * IQR from the hinge, the lower whisker extends from the hinge to the smallest value at most 1.5 * IQR from the hinge. Outliers are plotted individually. Wilcoxon tests were performed.


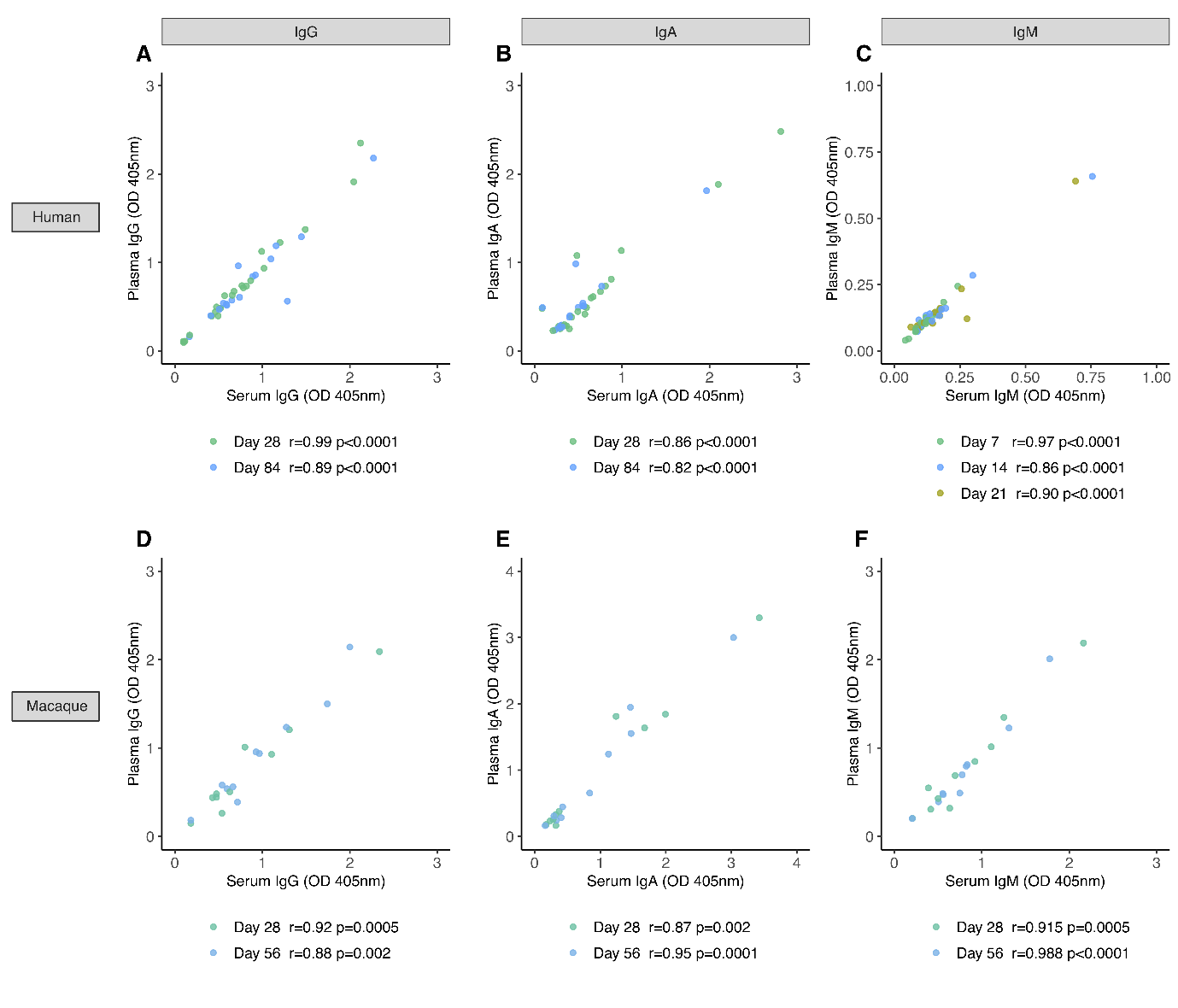
**Figure S6. PPD-specific antibody responses to BCG vaccination compared between plasma and serum.** Antibody responses in serum and plasma collected from healthy UK adults enrolled into human Study 1 (**A-C**) and rhesus macaques enrolled into macaque Study 1 (**D-F**). IgG (**A**) and IgA (**B**) were compared at 28 (green) and 84 (blue) days post-BCG vaccination in humans. IgM (**C**) responses were compared at 7 (green), 14 (blue) and 21 (khaki) days post-BCG vaccination. IgG (**D**), IgA (**E**) and IgM (**F**) responses were compared at 28 (green) and 56 (blue) days post-BCG vaccination in macaques. Spearman’s rank correlations were performed.


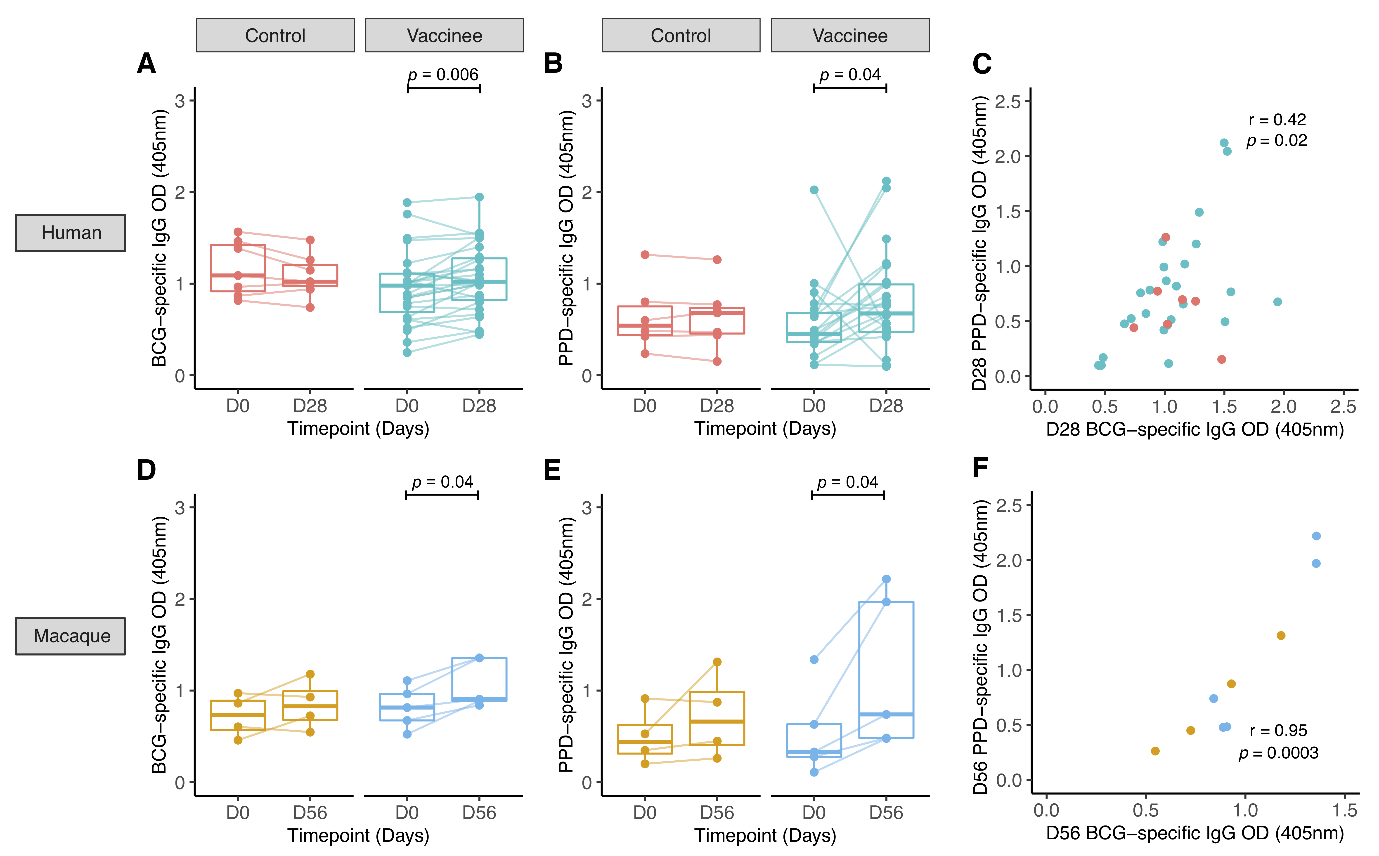


**Figure S7. BCG-specific and PPD-specific IgG responses to BCG vaccination in serum.** IgG responses in serum collected from healthy UK adults enrolled into human Study 1 (red = unvaccinated controls, cyan = BCG vaccinees, **A-C**), rhesus macaques enrolled into macaque Study 3 (yellow = unvaccinated controls, blue = BCG vaccinees, **D-F**). IgG responses were compared to either whole BCG (**A, D**) or to PPD (**B, E**), and the association between the two was determined (**C, F**). Points represent the mean of triplicate values, boxes indicate the median value with the interquartile range (IQR) and the upper whisker extends to the largest value no further than 1.5 * IQR from the hinge, the lower whisker extends from the hinge to the smallest value at most 1.5 * IQR from the hinge. Outliers are plotted individually. A Wilcoxon test was used to compare between time-points in the BCG vaccinated groups (**A, B, D, E**), and a Spearman’s rank correlation was used to determine associations (**C, F**).


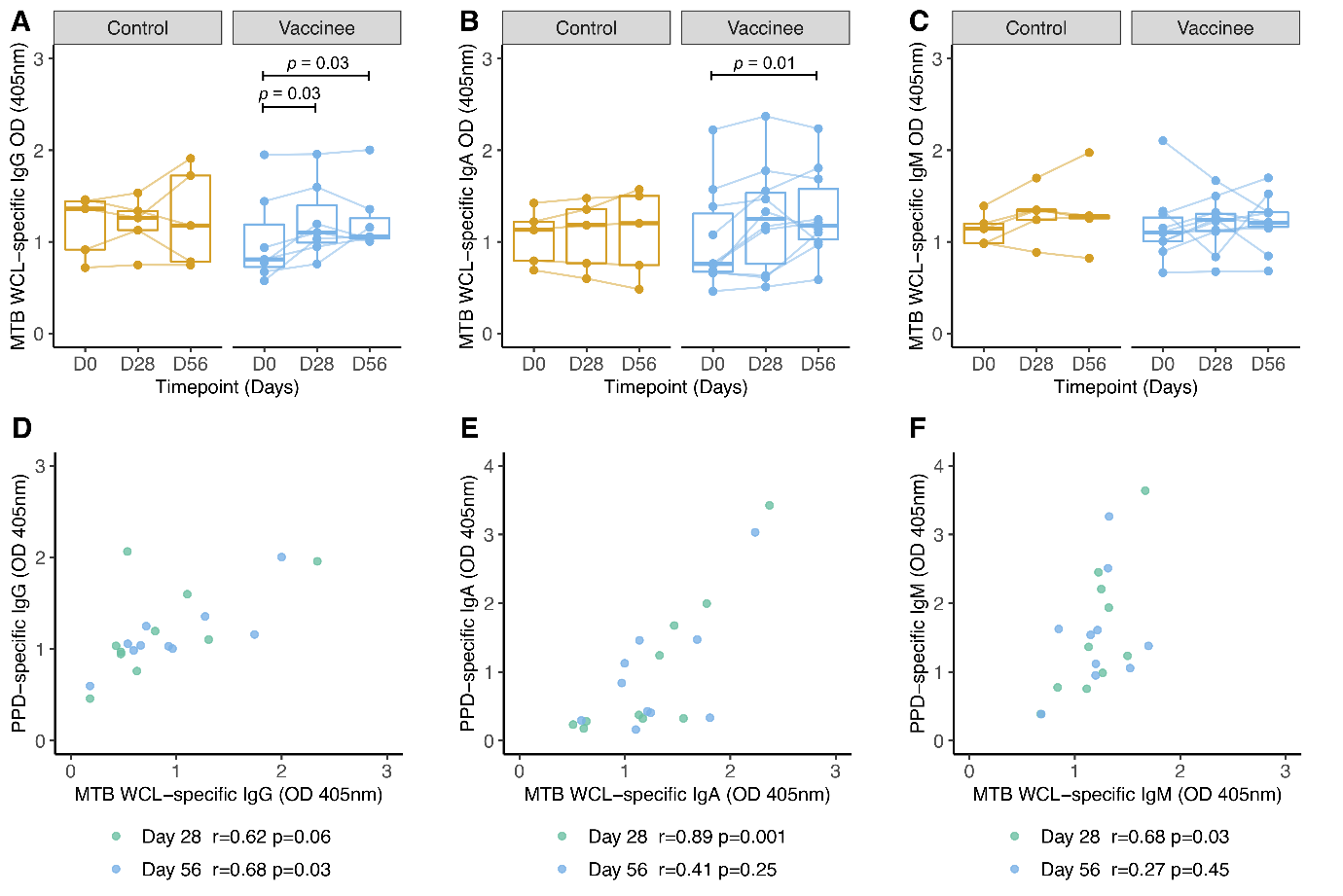


**Figure S8. MTB WCL-specific antibody responses to BCG vaccination in rhesus macaques.** Serum was collected from animals enrolled into macaque Study 1 which were either unvaccinated controls (yellow) or received BCG vaccination (blue). MTB WCL-specific IgG (**A**), IgA (**B**) and IgM (**C**) responses were measured in serum over time, and associations determined between responses following BCG vaccination to MTB WCL and PPD for IgG (**D**), IgA (**E**) and IgM (**F**). Points represent the mean of triplicate values, boxes indicate the median value with the interquartile range (IQR) and the upper whisker extends to the largest value no further than 1.5 * IQR from the hinge, the lower whisker extends from the hinge to the smallest value at most 1.5 * IQR from the hinge. Outliers are plotted individually. A Friedman test with Dunn’s correction for multiple comparisons was used to compare the BCG vaccine-induced response between post-vaccination and baseline time-points (**A-C**), and a Spearman’s rank correlation was used to determine associations (**D-F**).


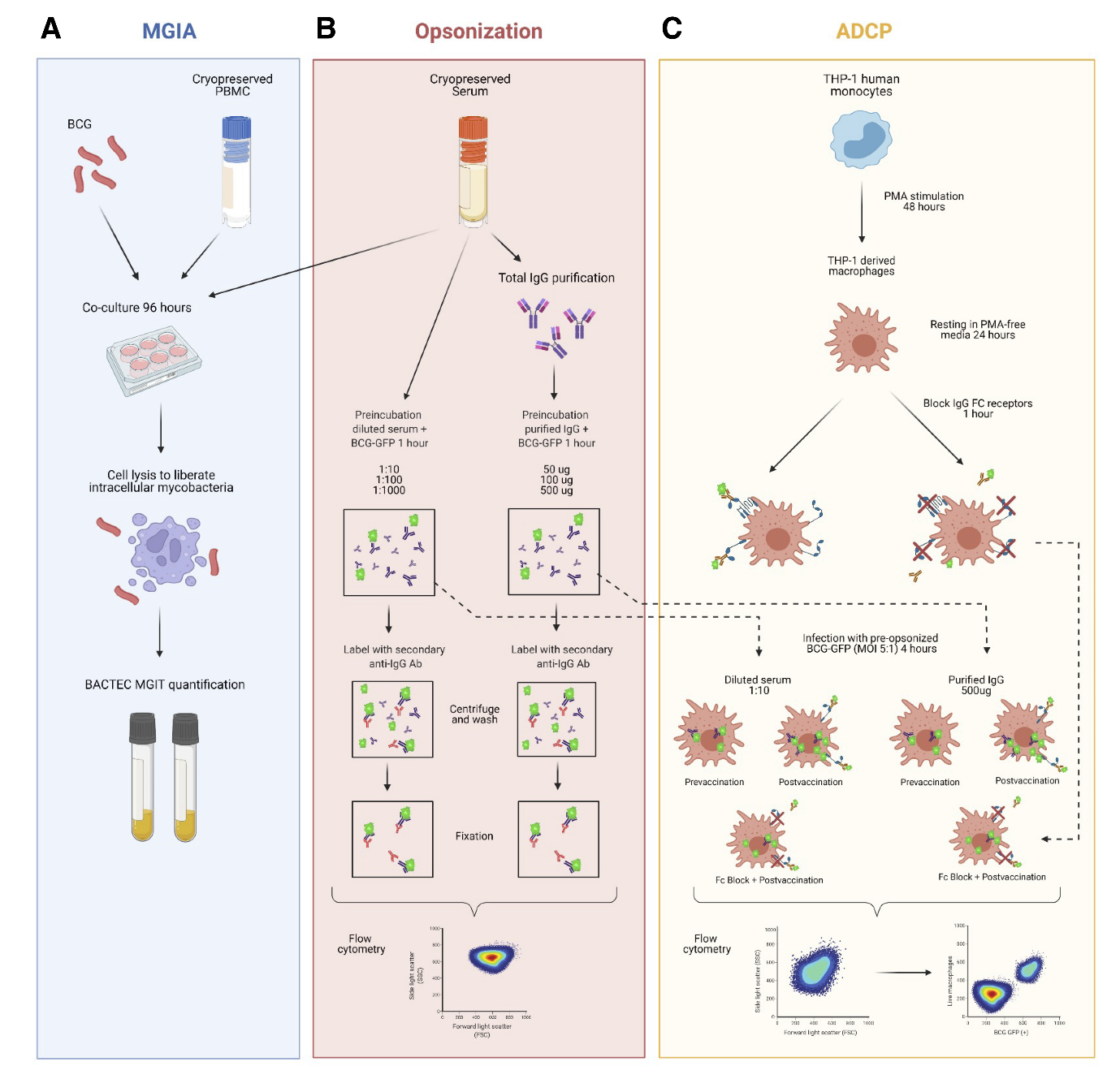


**Figure S9. Workflow for functional assays.** MGIA (**A**), Opsonization (**B**) and ADCP (**C**) assays were performed on samples taken from human Study 1.


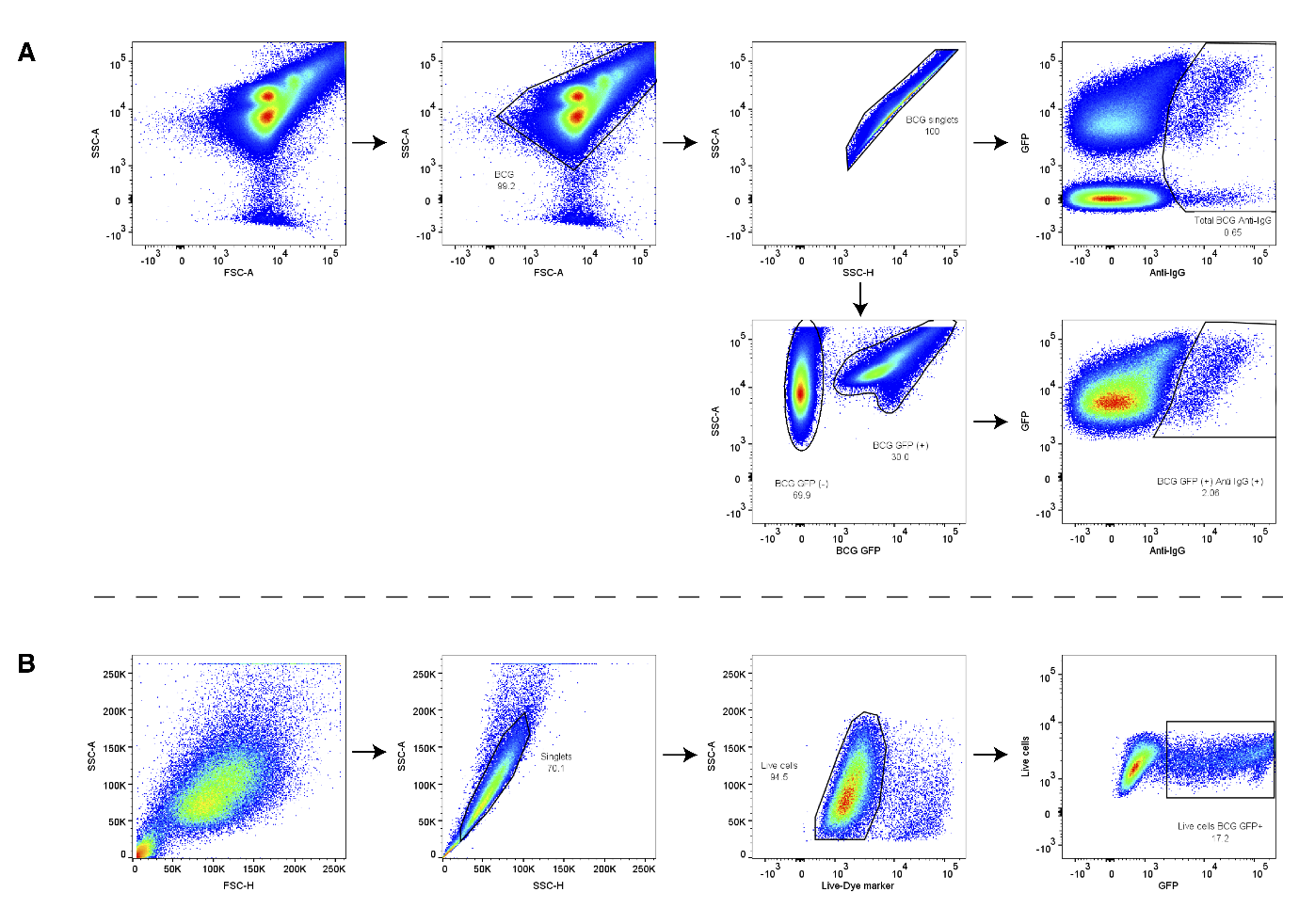
**Figure S10. Flow cytometry gating strategies**. For the opsonization assay, cells were gated on total events, singlets, and then anti-IgG+ total BCG, or anti-IgG+ GFP+ BCG (**A**). For the ADCP assay, cells were gates on total events, singlets, and then BCG-GFP+ live cells (**B**).


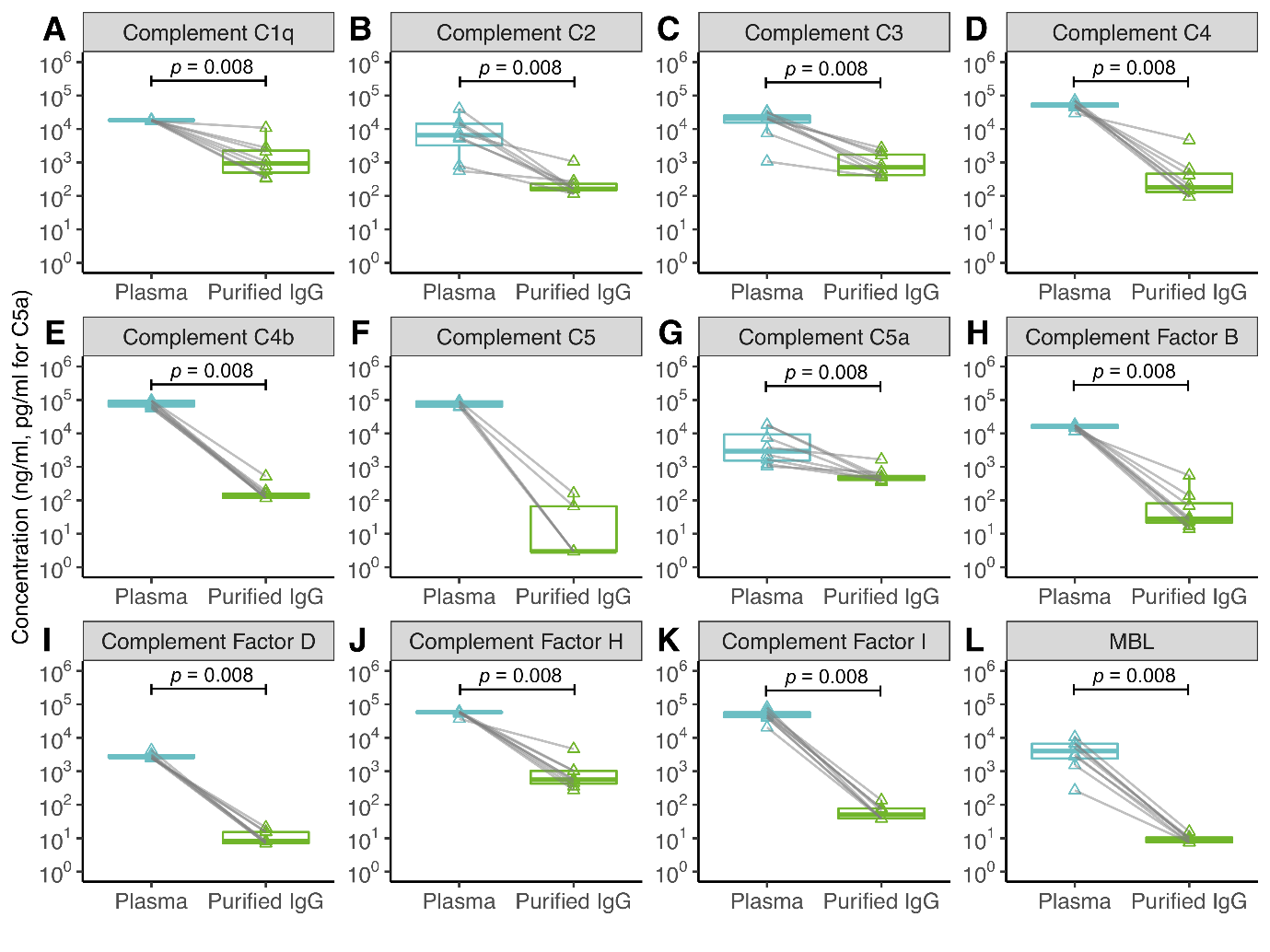
**Figure S11. Confirmation of complement depletion by Luminex.** Samples were used from n=9 healthy UK adults enrolled into human Study 1, all of whom received ID BCG vaccination. IgG was purified from plasma by affinity chromatography and the concentration of complement proteins C1q (**A**), C2 (**B**), C3 (**C**), C4 (**D**), C4b (**E**), C5 (**F**), C5a (**G**), Factor B (**H**), Factor D (**I**), Factor H (**J**), Factor I (**K**) and Mannan-binding lectin (MBL) (**L**) were measured by Luminex in whole plasma (cyan) and the purified IgG fraction (green). Points represent the mean of triplicate values, boxes indicate the median value with the interquartile range (IQR) and the upper whisker extends to the largest value no further than 1.5 * IQR from the hinge, the lower whisker extends from the hinge to the smallest value at most 1.5 * IQR from the hinge. Outliers are plotted individually. Wilcoxon tests were performed.


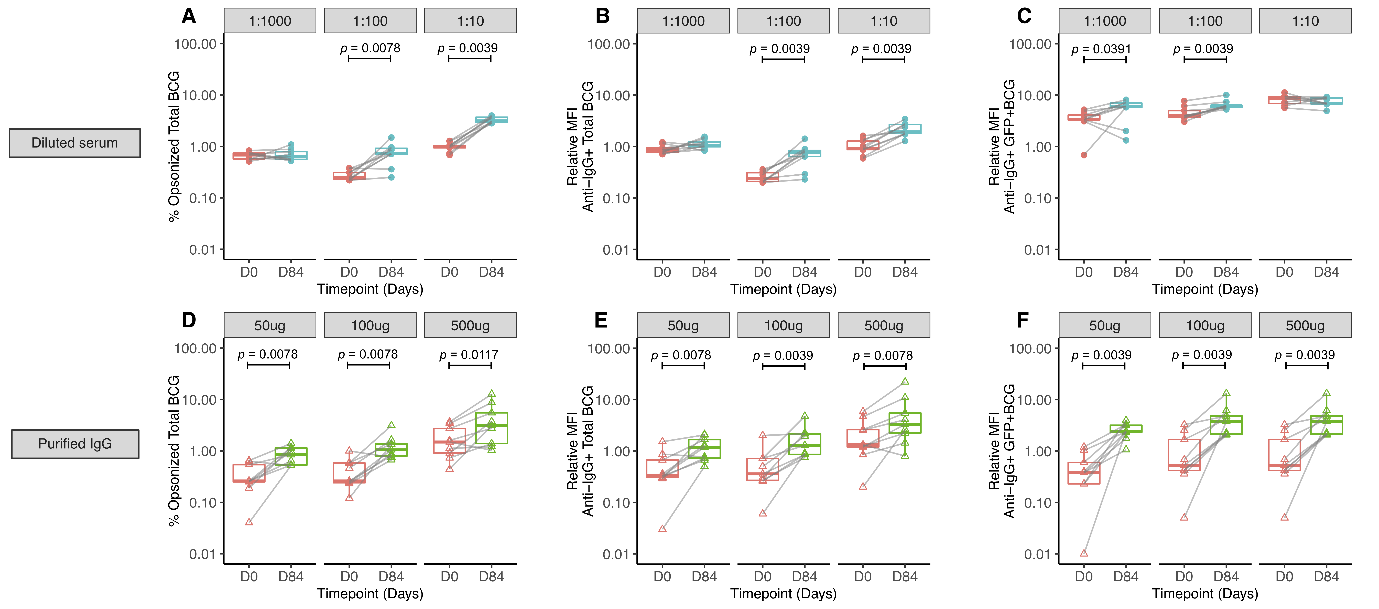


**Figure S12. Opsonization assay data by different gating strategies.** Samples were used from n=9 healthy UK adults enrolled into human Study 1, all of whom received ID BCG vaccination. BCG-GFP was incubated with either diluted serum (**A-C**) or purified IgG (**D-F**) from baseline and 84 days post-BCG vaccination and the percentage of BCG-GFP (**C, F**) or total BCG including both GFP+ and GFP- (**A, B, D, E**) that was opsonized was determined by flow cytometry. Results are presented as either percentage of opsonized BCG (**A, D**) or relative MFI (median fluorescence intensity divided by the number of anti-IgG+ events detected) (**B, C, E, F**). Points represent the mean of triplicate values, boxes indicate the median value with the interquartile range (IQR) and the upper whisker extends to the largest value no further than 1.5 * IQR from the hinge, the lower whisker extends from the hinge to the smallest value at most 1.5 * IQR from the hinge. Outliers are plotted individually. Wilcoxon tests were performed.

**2.0 Supplementary tables**

|  |  | **IgG** | | **IgA** | | **IgM** | | |
| --- | --- | --- | --- | --- | --- | --- | --- | --- |
|  |  | **D28 FC** | **D84 FC** | **D28 FC** | **D84 FC** | **D7**  **FC** | **D14 FC** | **D21 FC** |
| **IgG** | **D28 FC** |  | .846  .000  *** | .667  .003  ** | .824  .001  ** | .279  .315 | .527  .096 | .358  .310 |
|  | **D84 FC** | .846  .000  *** |  | .495  .086 | .697  .006  ** | -.005  .986 | .286  .535 | .750  .052 |
| **IgA** | **D28 FC** | .667  .003  ** | .495  .086 |  | .609  .021  * | .015  .958 | .373  .259 | .248  .489 |
|  | **D84 FC** | .824  .001  ** | .697  .006  ** | .609  .021  * |  | .308  .331 | .679  .094 | .383  .383 |
| **IgM** | **D7 FC** | .279  .315 | -.005  .986 | .015  .958 | .308  .331 |  | .588  .074 | -.450  .224 |
|  | **D14 FC** | .527  .096 | .286  .535 | .373  .259 | .679  .094 | .588  .074 |  | .383  .308 |
|  | **D21 FC** | .358  .310 | .750  .052 | .248  .489 | .383  .383 | -.450  .224 | .383  .308 |  |

**Table S1.** Spearman’s correlations between fold change (post-vaccination/baseline) in different isotypes following BCG vaccination in serum collected from healthy UK adults enrolled in human Study 1.

|  |  | **IgG** | | **IgA** | | **IgM** | |
| --- | --- | --- | --- | --- | --- | --- | --- |
|  |  | **D28 FC** | **D56 FC** | **D28 FC** | **D56 FC** | **D28 FC** | **D56 FC** |
| **IgG** | **D28 FC** |  | .648  .043  * | .903  .000  *** | .661  .038  * | .697  .025  * | .709  .022  * |
|  | **D56 FC** | .648  .043  * |  | .552  .098 | .988  .000  ** | .600  .067 | .758  .011  * |
| **IgA** | **D28 FC** | .903  .000  *** | .552  .098 |  | .564  .090 | .648  .043  * | .503  .138 |
|  | **D56 FC** | .661  .038  * | .988  .000  ** | .564  .090 |  | .539  .108 | .782  .008  ** |
| **IgM** | **D28 FC** | .697  .025  * | .600  .067 | .648  .043  * | .539  .108 |  | .600  .067 |
|  | **D56 FC** | .709  .022  * | .758  .011  * | .503  .138 | .782  .008  ** | .600  .067 |  |

**Table S2.** Spearman’s correlations between fold change (post-vaccination/baseline) in different isotypes following BCG vaccination in serum collected from rhesus macaques enrolled in macaque Study 1.

|  |  | **IgG** | | **IgA** | | **IgM** | | |
| --- | --- | --- | --- | --- | --- | --- | --- | --- |
|  |  | **D28 FC** | **D84 FC** | **D28 FC** | **D84 FC** | **D7**  **FC** | **D14 FC** | **D21 FC** |
| **IgG** | **D28 FC** |  | .748  .000  *** | .475  .040  * | .253  .297 | .271  .328 | .329  .297 | .103  .777 |
|  | **D84 FC** | .748  .000  *** |  | .269  .280 | .441  .052 | .138  .637 | .218  .519 | .224  .484 |
| **IgA** | **D28 FC** | .475  .040  * | .269  .280 |  | .444  .057 | .843  .000  *** | .762  .004  ** | .164  .651 |
|  | **D84 FC** | .253  .297 | .441  .052 | .444  .057 |  | .332  .226 | .224  .484 | .077  .812 |
| **IgM** | **D7 FC** | .271  .328 | .138  .637 | .843  .000  *** | .332  .226 |  | .736  .010  ** | .100  .798 |
|  | **D14 FC** | .329  .297 | .218  .519 | .762  .004  ** | .224  .484 | .736  .010  ** |  | .517  .154 |
|  | **D21 FC** | .103  .777 | .224  .484 | .164  .651 | .077  .812 | .100  .798 | .517  .154 |  |

**Table S3.** Spearman’s correlations between fold change (post-vaccination/baseline) in different isotypes following BCG vaccination in plasma collected from healthy UK adults enrolled in human Study 1.

|  |  | **IgG** | | **IgA** | | **IgM** | |
| --- | --- | --- | --- | --- | --- | --- | --- |
|  |  | **D28 FC** | **D56 FC** | **D28 FC** | **D56 FC** | **D28 FC** | **D56 FC** |
| **IgG** | **D28 FC** |  | .709  .022  * | .733  .016  * | .564  .090 | .842  .002  ** | .382  .276 |
|  | **D56 FC** | .709  .022  * |  | .394  .260 | .903  .000  *** | .479  .162 | .418  .229 |
| **IgA** | **D28 FC** | .733  .016  * | .394  .260 |  | .394  .260 | .648  .043  * | .576  .082 |
|  | **D56 FC** | .564  .090 | .903  .000  *** | .394  .260 |  | .261  .467 | .539  .108 |
| **IgM** | **D28 FC** | .842  .002  ** | .479  .162 | .648  .043  * | .261  .467 |  | .467  .174 |
|  | **D56 FC** | .382  .276 | .418  .229 | .576  .082 | .539  .108 | .467  .174 |  |

**Table S4.** Spearman’s correlations between fold change (post-vaccination/baseline) in different isotypes following BCG vaccination in plasma collected from rhesus macaques enrolled in macaque Study 1.
